# Supplementary material for: Dynamic characterization and interpretation for protein-RNA interactions across diverse cellular conditions using HDRNet
Source: Nat Commun. 2023 Oct 26;14:6824. doi: 10.1038/s41467-023-42547-1 (PMC10603054; doi:10.1038/s41467-023-42547-1)
Supplement: Supplementary file 1 — Supplementary Information [file 41467_2023_42547_MOESM1_ESM.pdf]

Supplementary Information "Dynamic characterization and interpretation for protein–RNA interactions across diverse cellular conditions using HDRNet"

Haoran Zhu[1], **Yuning Yang**[2], **Yunhe Wang**[3], **Fuzhou Wang**[4], **Yujian Huang**[5], **Yi Chang**[1], **Ka-chun Wong**[4,✉], **and Xiangtao Li**[1,✉]

[1]School of Artificial Intelligence, Jilin University, Changchun, 130012, China.
[2]Donnelly Centre for Cellular and Biomolecular Research, University of Toronto, Toronto, ON, Canada
[3]School of Artificial Intelligence, Hebei University of Technology, Tianjin, China.
[4]Department of Computer Science, City University of Hong Kong, Hong Kong SAR.
[5]College of Computer Science and Cyber Security, Chengdu University of Technology, Chengdu, 610059, China

Correspondence: ***kc.w@cityu.edu.hk;lixt314@jlu.edu.cn***

DRAFT

Index

1 HDRNet provides better performance than classic machine learning algorithms 3

2 Comparing icSHAPE with other in vivo and computationally predicted secondary structure profiles 5

3 RNA motif-finding/scanning strategy implemented in PRIESSTESS augment the performance of HDRNet. 7

4 Hierarchical Structure Improves the Prediction Performance and Feature Correlation of HDRNet 9

5 Existing sub-groups of binding events that are better characterized by HDRNet 10

6 Analyses of the specific features associated with FMR1 and FXR2 12

7 HDRNet has superior performance for RBPs with high and low expression levels and for target RNA events with high and low expression levels in different cellular contexts. 14

8 HDRNet predicts dynamic RNA-RBP interactions on in vivo tissues under normal and disease conditions. 17

9 Motifs identified by the dynamic global contextual embedding are highly comparable to known motifs. 20

10 HDRNet captured the binding peaks associated with the extracted motifs and identified the structural preferences of the binding events. 21

11 Visualization of the self-attention mechanism of the BERT model 22

12 HDRNet can detect high-attention binding region with variants allels. 23

13 Nucleotide mutations lead to potential RNA structural changes. 24

14 Protein-Protein interaction (PPI) analysis of the identified dynamic binding genes from TDP-43 binding transcripts 27

15 Pan-cancer analysis under the identified hub-genes 29

16 Hub gene-disease association network 31

17 Interaction network of disease-associated genes with TFs and miRNAs 32

18 Possible drug molecules for treatment of neurological disorders 34

19 The utilization of self-attention, BERT, and residual networks in the task of RBP binding sites predictions. 36

Supplementary Note 1: HDRNet provides better performance than classic machine learning algorithms

To further demonstrate the effectiveness of our proposed HDRNet, we compared HDRNet with seven machine learning algorithms, namely XGBoost (1), Random Forest (RF) (2), Logistic Regression (LR) (3), Artificial Neural Network (ANN) (4), ExtraTreeClassifier (ETC) (5), SGDClassifier (SGDC) (6), and GaussianNB (GNB) (7). These algorithms were implemented in the scikit-learn package (8). The experimental results are shown in Supplementary Fig. 1. From the comparison results, we have observed that HDRNet outperformed those machine learning algorithms across a substantial proportion of the RBP datasets in both static and dynamic prediction tasks. As illustrated in Supplementary Fig. 1a, HDRNet achieved the highest AUC in the static prediction task. It is worth noting that, among the compared machine learning algorithms, XGBoost exhibited superior performance, whereas ETC performed the worst. ANN also produced comparable results, likely due to the proficiency of the neural network framework for extracting features. However, ANN performed extremely poorly on certain RBP datasets, with an AUC of 0.5, whereas HDRNet achieved an AUC exceeding 0.8. On the other hand, as depicted in Supplementary Fig. 1b, HDRNet consistently outperformed all compared machine learning algorithms in dynamic prediction tasks. Notably, the dynamic prediction performance of ANN was degraded, compared to the static prediction performance. Overall, these results highlight the benefits and advantages of the proposed HDRNet architecture, as the neural network framework alone performs poorly in dynamic prediction.

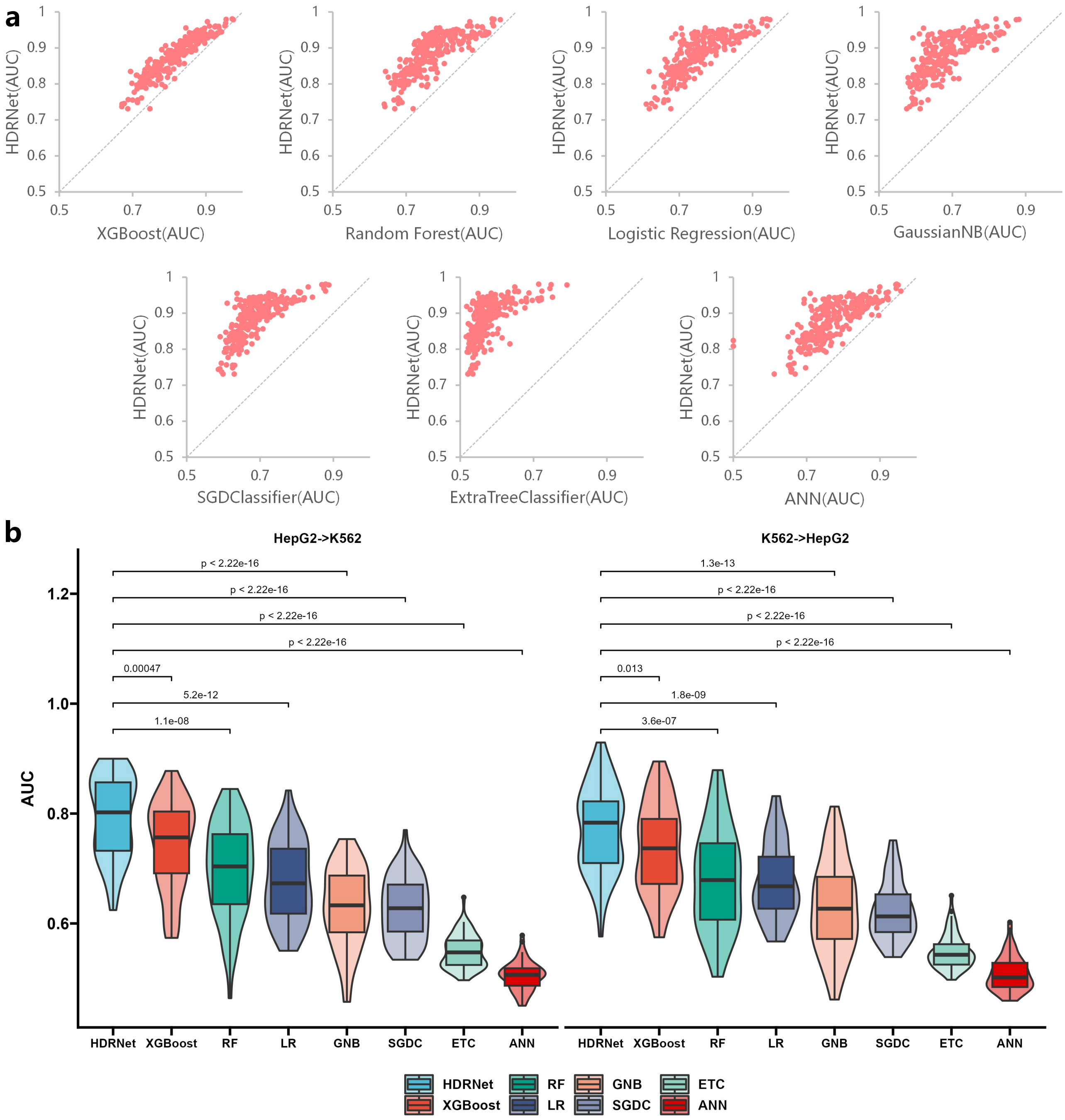

Supplementary Fig. 1. (a) Scatter plot comparing AUC scores of HDRNet with other machine learning algorithms. (b) Comparative analysis of the overall dynamic prediction performance between HDRNet and other machine learning methods (n=68 in each group; center line, median; box limits, upper and lower quartiles; whiskers, 1.5× interquartile range; Wilcox test). Source data are provided as a Source Data file.

Supplementary Note 2: Comparing icSHAPE with other in vivo and computationally predicted secondary structure profiles

To demonstrate the effectiveness of icSHAPE in our HDRNet model further, we conducted an experiment to compare icSHAPE with other RNA secondary structure representation models, namely RNAfold (9) and two in-vivo secondary structural characterization methods: DMS-seq (10) and DMS-MaPSeq (11). To ensure a fair comparison, we kept all other modules of HDRNet, substituting only icSHAPE with the structural features derived from the comparative methods. The experimental results are summarized in Supplementary Fig. 2. As depicted in Supplementary Fig. 2a, the HDRNet model with icSHAPE for structural representation outperformed HDRNet models with other structural features in identifying protein-RNA interactions. We speculate that computationally predicted secondary structures could be obsolete and hence prone to artifacts. In contrast, in-vivo secondary structure scores, representing the probabilities of nucleotide pairing, could potentially offer rich structural information. Taking the SLTM protein dataset from K562 cells as an example, we subsequently utilized t-SNE to visualize the embedding representation of HDRNet with various structural information. As illustrated in Supplementary Fig. 2b, icSHAPE demonstrated a distinct division between positive and negative samples, with each group clearly situated on opposite sides. However, for DMS-seq and DMS-MaPseq, positive and negative samples are considerably overlapped between the clusters, whereas the performance of RNAfold was deemed suboptimal due to insufficient sample separation.

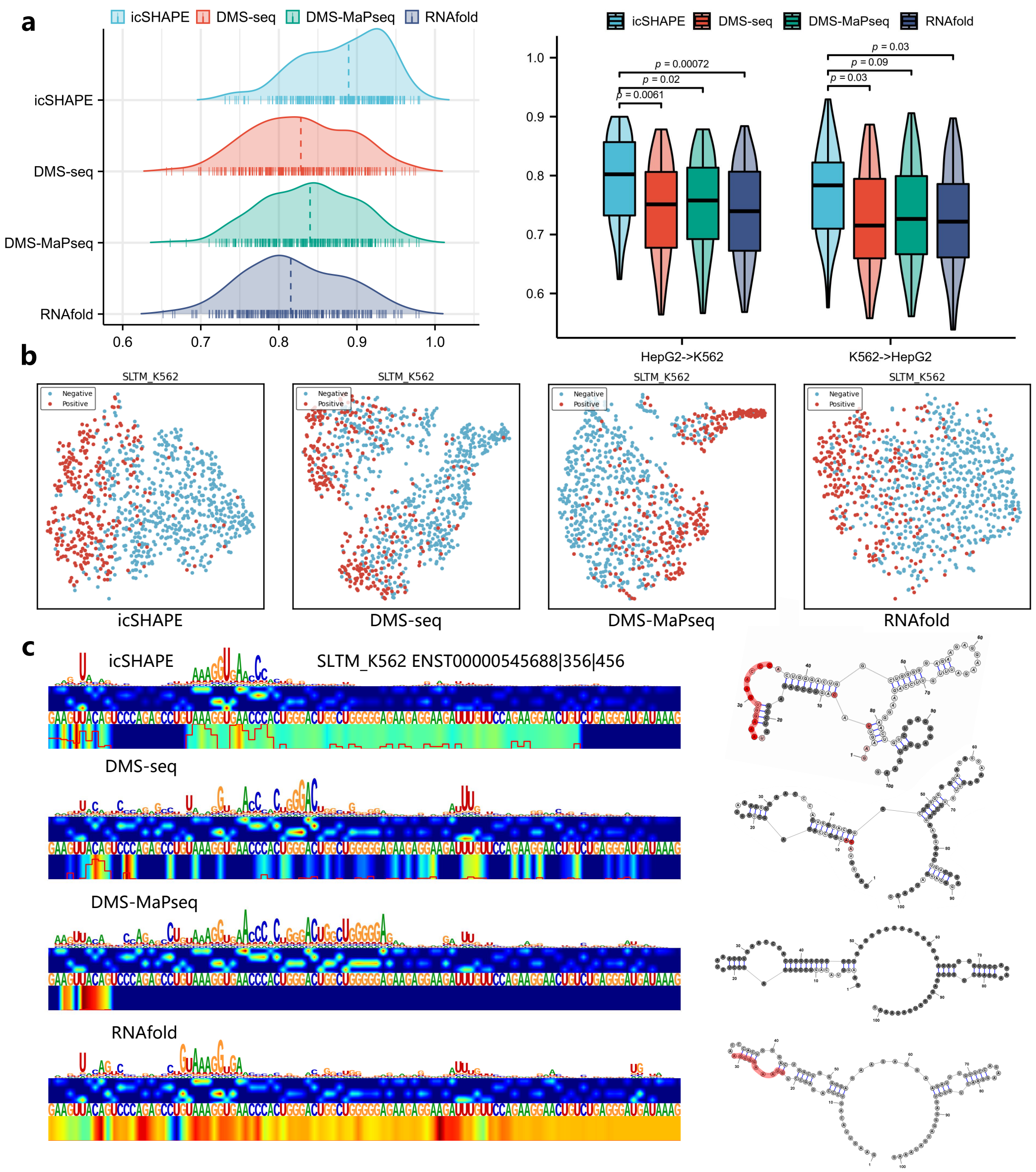

Supplementary Fig. 2. (a) Overall results of HDRNet using different structure information. HDRNet with icSHAPE performs best in both static and dynamic prediction tasks (n=68 in each group; center line, median; box limits, upper and lower quartiles; whiskers, 1.5× interquartile range; Dunn's test). (b) The latent embedding of the learned features by HDRNet using different types of input structure features. (c) Left: The high attention binding region identified by HDRNet with different input structure characterization. Right: The structural models predicted by RNAfold with the corresponding structural technology scores as constraints. Source data are provided as a Source Data file.

Supplementary Fig. 2c provides a visual representation of the high-attention binding regions identified by different RNA secondary structure representation models. Notably, icSHAPE and RNAfold, when used as the structural features within the HDRNet model, successfully captured continuous high-attention regions. Conversely, DMS-seq and DMS-MaPseq exhibited a considerable number of missing positions, resulting in a lack of continuity in the regions they identified. Moreover, the RNA models with those secondary structure features exhibited significant differences; for instance, icSHAPE could predict RNA models with greater structural heterogeneity and a higher number of nucleotide pairings. Conversely, RNAfold structures resembled those constrained using DMS-MaPseq, suggesting that the performance of DMS-MaPseq in structure prediction may be vulnerable to of missing positions. On this basis, we can conclude that these analyses not only highlight the effectiveness of icSHAPE features but also underscores potential limitations. Since the acquisition of icSHAPE data relies on sequencing experiments, RNAfold or other secondary structure prediction algorithms may serve as suitable alternatives for general tasks. These observations emphasize the importance of carefully selecting the most appropriate RNA secondary structure representation model based on the specific requirements of the task at hand.

We also conducted further experiments to investigate and compare the performance of HDRNet by integrating RNA structure information from different methods. Specifically, we explored multiple secondary structure features obtained from different methods and then employed a concatenation strategy to integrate them into the HDRNet model. As demonstrated in Supplementary Fig. 3, our observations indicate that the performance of HDRNet was not further improved but slightly decreased by integrating other secondary structure features. This observation indicates that those in vivo secondary structure information provides a more accurate depiction of the RNA environment than the computationally predicted secondary structure information (RNAfold), thus ensuring the performance of HDRNet. These results support the reliability of HDRNet and suggest that the original HDRNet is sufficiently robust as a standalone model.

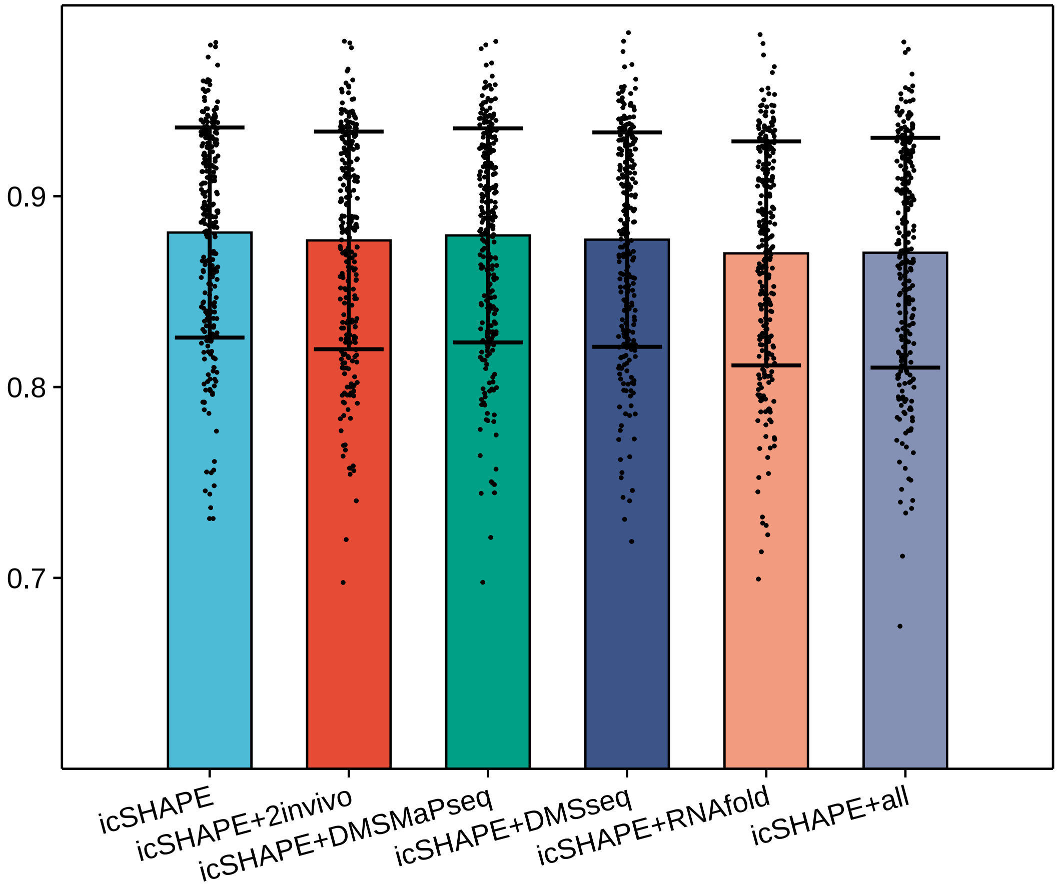

Supplementary Fig. 3. Ablation comparison results of integrating RNA structure information from different methods. It can be observed that combining other features didn't further improve the performance of HDRNet (n=261 in each group, with mean $\pm$ SD). Source data are provided as a Source Data file.

Supplementary Note 3: RNA motif-finding/scanning strategy implemented in PRIESSTESS augment the performance of HDRNet.

In the original study, PRIESSTESS was introduced as a universal RNA motif-finding/scanning strategy capable of identifying enriched RNA sequence and/or structure motifs. To incorporate the PRIESSTESS feature representation into our model, we employed a step-by-step approach. First, we used RNAfold to predict the secondary structure of each sequence and annotated them using the seven alphabets annotation described in PRIESSTESS. Then, we utilized the STREME tool to extract enriched motifs within each alphabets annotation, generating Position Frequency Matrices (PFMs) for each motif. For each annotation, we scanned of the obtained PFMs on the data. To create an augmentation of HDRNet, we calculated the sum of the top four scores per sequence for each PFM. This augmentation, derived mainly from the sequence-structure motifs, was integrated into the icSHAPE feature to enhance the expression of the structure profiles. Specifically, we employed a fully connected network to adjust the feature length to match with that of icSHAPE and then summed them together.

After that, we conducted experiments to verify whether the augmented features can further impact the performance of HDRNet. As depicted in Supplementary Fig. 4, our results demonstrate that the average performance of HDRNet after feature enhancement was equal to the original features in static prediction task and showed a very slight decrease in the dynamic prediction task, as illustrated in Supplementary Fig. 5. However, it is worth noting that we observed significant improvements in the performance of the model after data augmentation on certain datasets. For instance, in the C17ORF85_HEK293 and TNRC6A_HEK293 datasets, the performance of static prediction was enhanced by 2.2% and 2.4%, respectively. Additionally, we also observed a 2% performance improvement in the dynamic prediction task on the DDX52 dataset. These findings support the notion that the motif extraction and scoring based on the multi-sequence-structure annotation approach utilized by PRIESSTESS significantly contribute to the identification of RNA-binding protein (RBP) binding sites and can be an effective approach to enhance the performance of HDRNet when the binding site motif patterns of interest are well-conserved.

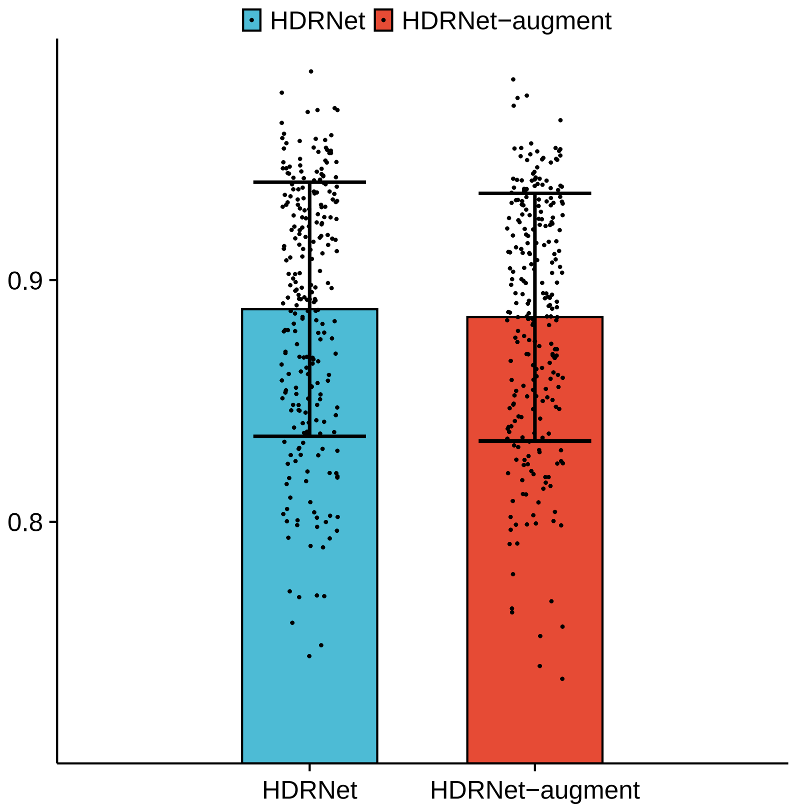

Supplementary Fig. 4. Performance comparison of HDRNet and HDRNet with feature augmentation on static prediction task (n=261 in each group, with mean ± SD). Source data are provided as a Source Data file.

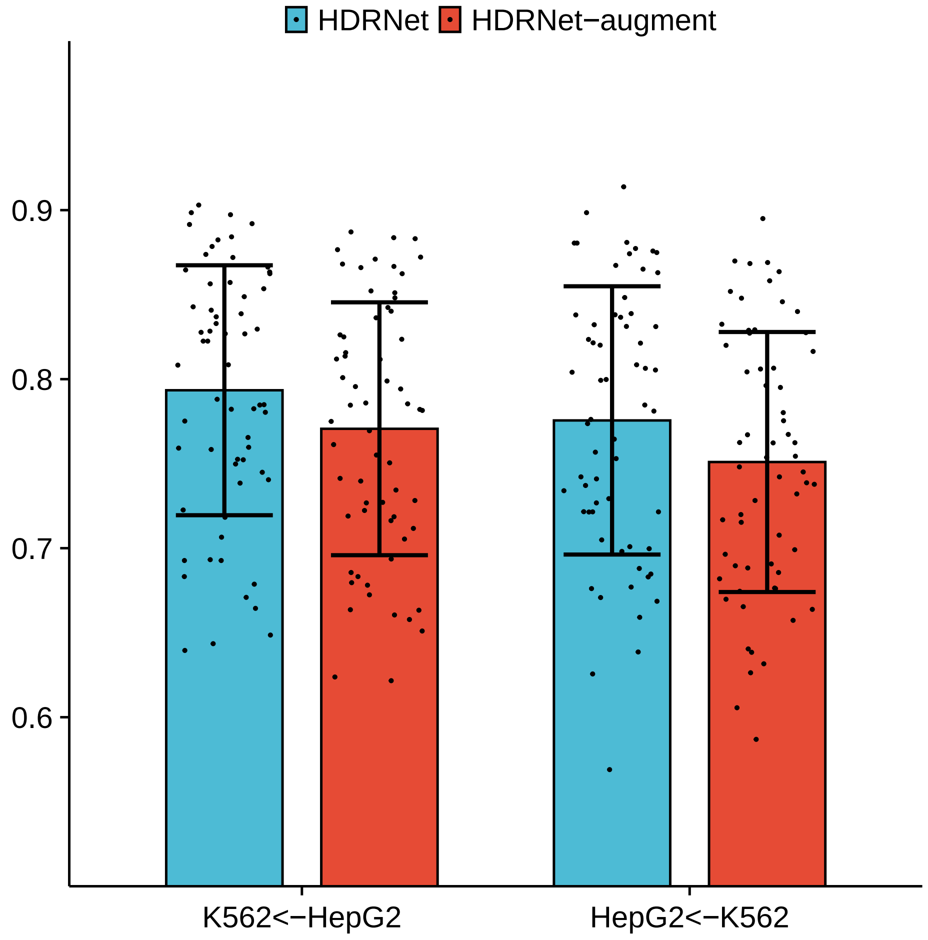

Supplementary Fig. 5. Performance comparison of HDRNet and HDRNet with feature augmentation on dynamic prediction task (n=62 in each group, with mean $\pm$ SD). Source data are provided as a Source Data file.

Supplementary Note 4: Hierarchical Structure Improves the Prediction Performance and Feature Correlation of HDRNet

To demonstrate the effectiveness of our chosen network architecture, we initially constructed a non-hierarchical HDRNet, referred to as HDRNet-nonhier, which combined multi-source features at the network's inception. After that, a comparative analysis was conducted between our proposed HDRNet and HDRNet-nonhier, evaluating their performance in both static and dynamic prediction tasks. The comparative results are summarized in Supplementary Fig. 6a, revealing that the HDRNet, characterized by its hierarchical structure, exhibited significantly superior prediction performance compared to its non-hierarchical counterpart (HDRNet-nonhier). Moreover, HDRNet, with its hierarchical architecture, demonstrated superior performance in intercellular dynamic prediction. Furthermore, we performed feature correlation analysis and visualization across different versions of HDRNet. For illustration purpose, we employed the TBRG4 protein dataset in HepG2 cells as a representative sample, as depicted in Supplementary Fig. 6b. We can observe that HDRNet with a hierarchical structure has a more distinct feature correlation and hierarchy than the non-hierarchical one. Therefore, sequence information and secondary structure profiles can be normalized and fused, enriching the features learned by HDRNet to be robust.

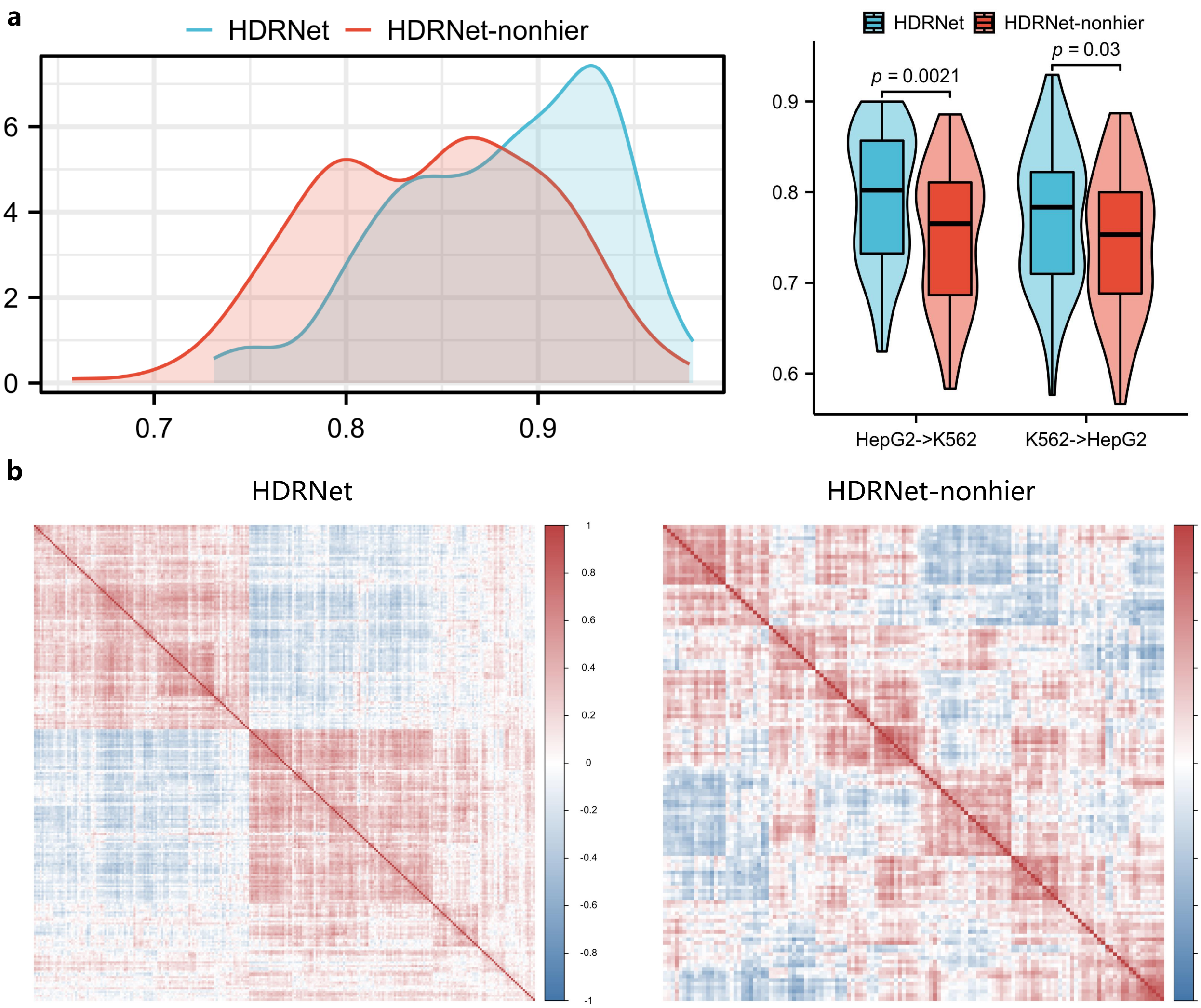

Supplementary Fig. 6. (a) Performance comparison of HDRNet with different hierarchical structures. The results demonstrate that the proposed hierarchical network achieves superior performance in both static and dynamic prediction tasks. (n=68 in each group; center line, median; box limits, upper and lower quartiles; whiskers, 1.5× interquartile range; T test.) (b) Feature correlation analysis of different HDRNet versions. Notably, HDRNet exhibits substantial feature correlation and feature hierarchy. Source data are provided as a Source Data file.

Supplementary Note 5: Existing sub-groups of binding events that are better characterized by HDRNet

In this part, we conducted a comparison between HDRNet and PrismNet, focusing on their performance across various cell lines, and the detailed results of this comparison are presented in Supplementary Fig. 7a. As demonstrated in this figure, we can observe that HDRNet was consistently superior to PrismNet in all cell lines evaluated, with significant enhancement observed in HEK293, HepG2, and K562 cells. To ensure the credibility of our findings, we selected datasets from these three cell lines in which HDRNet was at least 5% better than PrismNet. We then analyzed the performance gap between the proposed HDRNet and PrismNet models for these datasets, as demonstrated in Supplementary Fig. 7b. We found that HDRNet showed a more significant performance improvement in predicting multiple datasets in HEK293 cell line. For illustration purpose, we further explored those RBPs in HEK293 cell lines. To investigate it, we have mapped these RBPs into STRING (12) and then used the the Markov Cluster Algorithm (MCL) with an inflation rate of 2.5 to cluster them, as demonstrated in Supplementary Fig. 7c. As can be seen from the figure, RBPs with similar biological functions are clustered together, while RBP datasets exhibiting significant performance improvements are consistently grouped into the same functional clusters, e.g., 15% for FMR2 and 13% for FXR1, in the subclusters represented by the red nodes highlighted. Moreover, in this cluster, previous studies have demonstrated that several RBPs (e.g., FXR2, FMR1, LIN28B) have the coexpression in the same tissues (13), which further indicates that these RBPs in the cluster may have similar context or structures.

With the distinct subgroups identified, we proceeded with an analysis of the structural complexity of these RBPs. Initially, we utilized HDRNet to scan the RBP datasets and extracted the high-attention 6-mer fragments that HDRNet identified most frequently. Similar to PrismNet, we then calculated the structural complexity of these 6-mer fragments, generated the PWM matrix and represented their structural motifs where the structure component using the labels “U” for unpaired nucleotide and “P” for paired. As shown in Supplementary Fig. 7d, we observe that RBPs in the same subgroup show structural similarities. For instance, the subgroup of NUDT21, CPSF2, and CPSF4 exhibited a prevalent preference for binding to paired structures, whereas RBPs in the subgroup containing LIN28A demonstrated a higher tendency to interact with complex structure fragments. Meanwhile, the binding region of FBL usually does not pair. These phenomena demonstrate the potential structural differences between different subgroups.

To investigate context variations further, we explored the context variations between RBP clusters using 3-mer analysis, tailored to the tokenization used by our dynamic global contextual embedding approach. In specific, we calculated the relative content of each 3-mer token within each RBP dataset. Subsequently, we employed hierarchical clustering to group RBPs based on their 3-mer token content, thereby assigning RBPs with similar profiles to the same cluster. As shown in Supplementary Fig. 7e, it can be seen that the results are in close agreement with the clusters we obtained through the STRING database; for instance, RBPs such as FMR1, FXR2, LIN28A, and LIN28B were consistently assigned to the same cluster, while CPSF2 and CPSF4 were in a distinct cluster. These findings further support the notion that the 3-mer token with contextual information can serve as an informative feature to identify different sub-groups of RBPs.

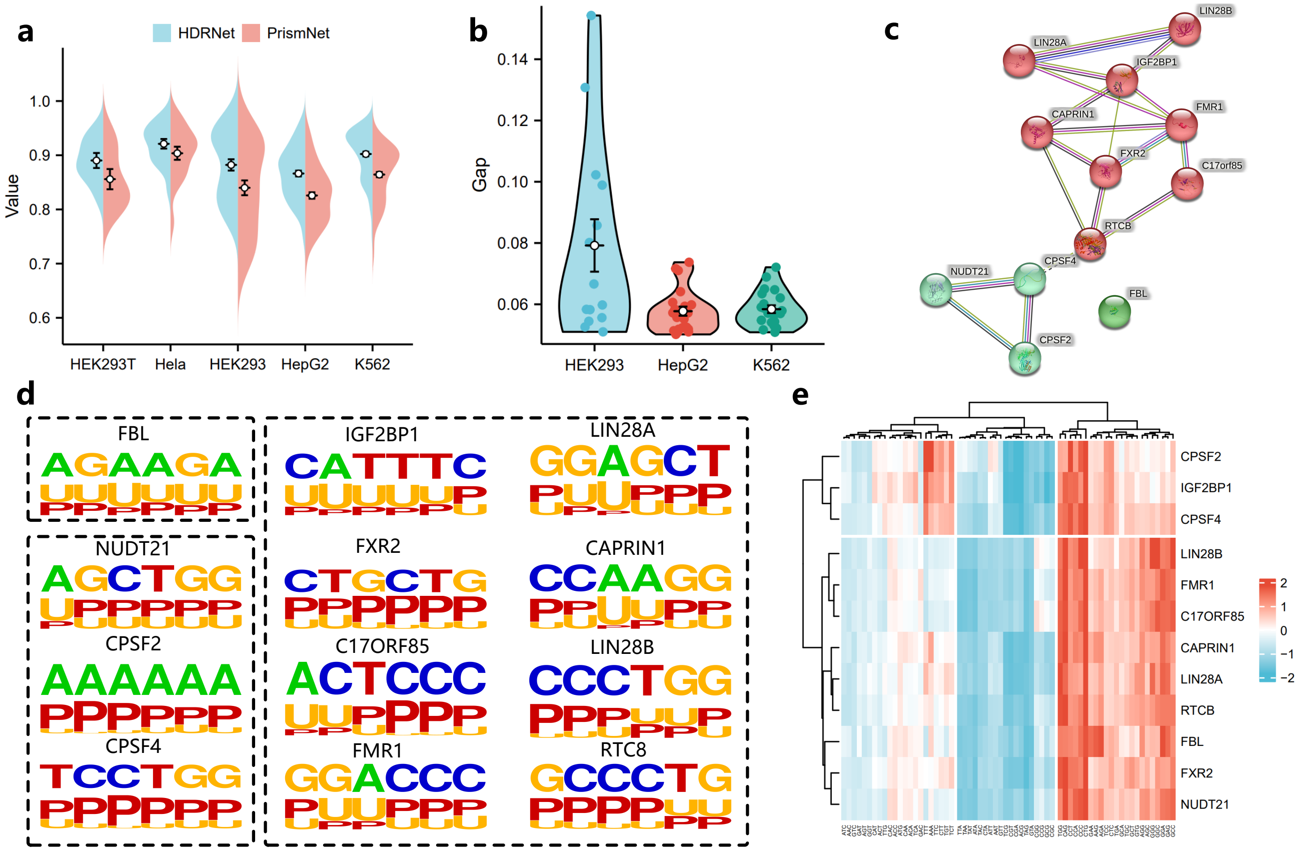

Supplementary Fig. 7. (a) Performance comparison of HDRNet and PrismNet through different cell lines (n=8, 17, 39, 84 and 112 in each group, with mean $\pm$ SD). (b) Performance gap between HDRNet and PrismNet through HEK293, HepG2 and K562 cell lines (n=17, 22 and 21 in each group, with mean $\pm$ SD). (c) Identified subgroups of RBP datasets in HEK293 cell line. (d) The integrative motifs identified by HDRNet on the RBPs within different subgroups. The top half presents the high-attention 6-mer fragment identified by HDRNet most frequently, while the lower half displays the structural motifs of the 6-mer sequence, where 'P' stands for paired, and 'U' indicates unpaired. (e) Heatmap representing the relative content of each 3-mer in the RBP binding site. RBPs with similar relative contents were grouped into a cluster by hierarchical clustering. Source data are provided as a Source Data file.

Supplementary Note 6: Analyses of the specific features associated with FMR1 and FXR2

Considering the intrinsic properties of FMR1 and FXR2 proteins, as previously mentioned, both FMR1 and FXR2 are members of the fragile X mental retardation protein (FMRP) family and are co-expressed in the cytoplasm of specific differentiated neurons (14). Despite being encoded by different genes located on chromosome X and chromosome 17, FMR1 and FXR2 share considerable similarity, with 60% sequence identity. Moreover, they display overlapping tissue distribution and have been found to interact *in vivo* (15). Additionally, FMRP proteins exhibit substantial functional overlap in terms of translational regulation and RNA-binding specificity (16), suggesting functional homology between FMR1 and FXR2 (17).

Furthermore, we investigated the potential properties of FMR1 and FXR2 in the context of RBP binding motifs. Firstly, we focused on the 6-mer distribution of the binding sites of FMR1 and FXR2 across different cell lines. As detailed in Supplementary Fig. 8a, we identified the top ten 6-mers with the highest enrichment in each dataset. Notably, the content of these 6-mers demonstrated remarkable similarities, with CAGCAG being highly represented across datasets. Moreover, a predominance of AG or CUG components within these 6-mers was noted, suggesting potential roles of AG and CUG in the binding domains of FMR1 and FXR2.

To further investigate the binding characteristics of FMR1 and FXR2, we applied HDRNet to scan these datasets and tabulated the 6-mer tokens with the maximum attention score in each sequence. The top ten high-attention 6-mers, most frequently identified by HDRNet, are depicted in Supplementary Fig. 8b. This analysis revealed a consensus high-attention 6-mer, CATGGA, in FMR1 datasets, while CTGCTG was consistently found across FXR2 datasets. Noteworthy observations include the presence of AGAAGA in the FMR1 dataset and AAGAAG in the FXR2 dataset, corroborating the binding motif previously suggested in (18). For a more precise visualization of binding properties, salient regions flagged by HDRNet are depicted in Supplementary Fig. 8c, where AG-rich and CUG-rich regions are highlighted within FMR1 and FXR2 datasets respectively. Interestingly, within the FMR1_HEK293 dataset, HDRNet accentuated the GGA segment, in spite of the prominence of the CATGGA 6-mer. Simultaneously, in the FXR2_HepG2 dataset, along with the underlined CUG region, HDRNet discerned the AAGAAG motif. Correspondingly, the attention distribution derived from the dynamic global contextual representation model further highlighted the AG-rich regions, as depicted in Supplementary Fig. 8d, where the constituent tokens comprise AGAAGA, coinciding with the motif we previously identified. This demonstrates the preferential focus of HDRNet on the regions enriched with AG, suggesting a potentially significant role of these AG-rich regions in the RNA binding process of FMR1 and FXR2.

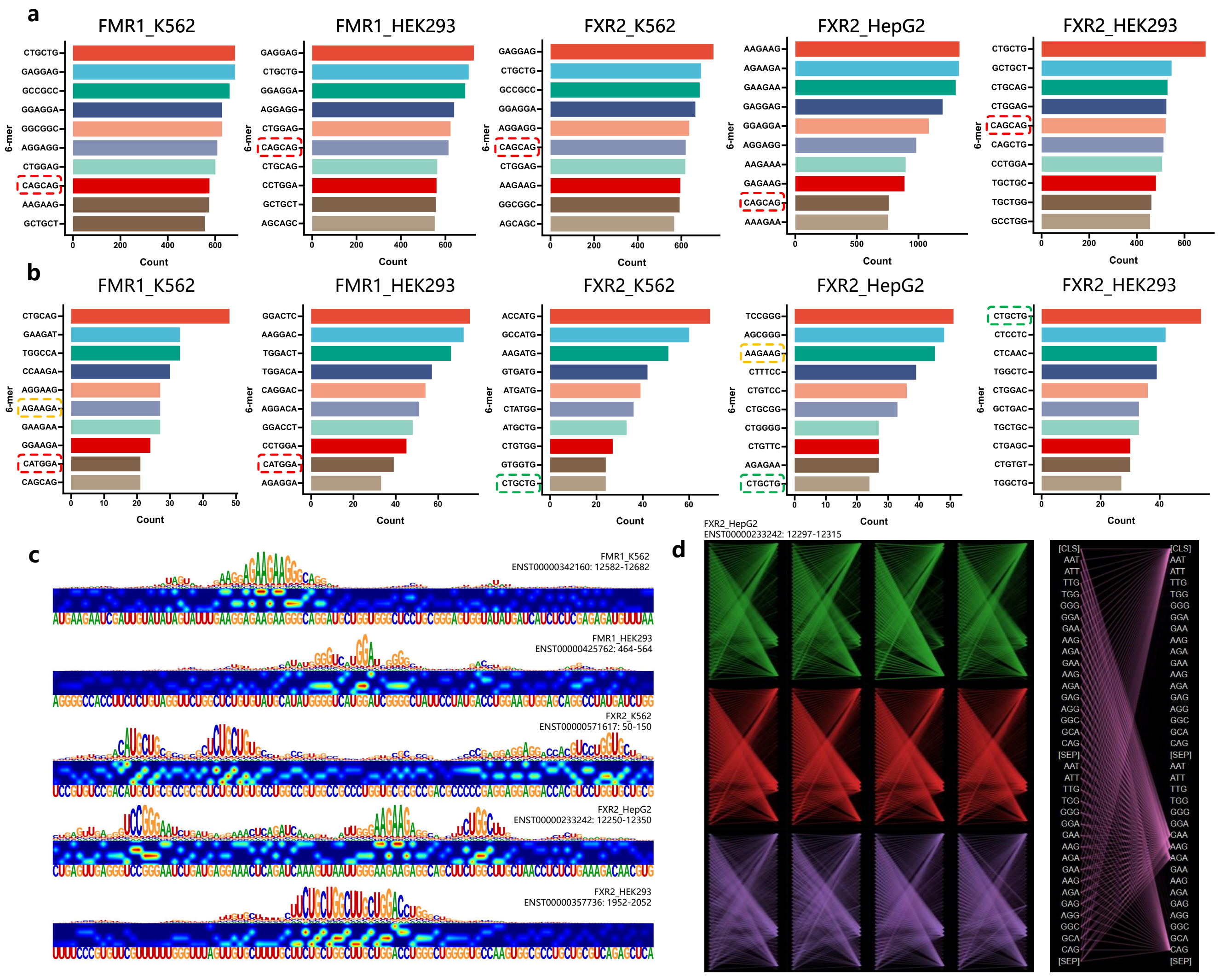

Supplementary Fig. 8. (a) Bar chart of 6-mer contents for FMR1 and FXR2 across different cell lines. (b) Statistics of the most significant 6-mer regions identified by HDRNet on the FMR1 and FXR2 datasets. (c) Visualization of the salient map detected by HDRNet, highlighting specific binding regions within the FMR1 and FXR2 datasets. (d) Visualization of attention distribution using dynamic global contextual embedding, with attention concentrated in A/G-rich regions. Source data are provided as a Source Data file.

Supplementary Note 7: HDRNet has superior performance for RBPs with high and low expression levels and for target RNA events with high and low expression levels in different cellular contexts.

To observe the validity of HDRNet across various expression levels of RBPs in both the same and different cellular contexts, we utilized the expression levels of RBPs in HepG2 and K562 cell lines from the reference (19) published in *Nature*, which provides a comprehensive collection of human RBPs in K562 and HepG2 cells from the Encyclopedia of DNA Elements (ENCODE) project phase III, including the expression levels of RBPs across different cell lines within the eCLIP dataset. To distinguish between high and low expression levels of RBPs, we employed the average expression level as the threshold. RBPs with expression levels surpassing this threshold were classified as highly expressed, whereas those below were considered lowly expressed. On this basis, we compared the performance of HDRNet with baseline methods separately on K562 and HepG2 cell lines. As illustrated in Supplementary Fig. 9a, we observed that HDRNet exhibited superior static prediction performance for RBPs with varying expression levels in different cell lines. Furthermore, we noted a slight improvement in HDRNet's performance on RBPs with higher expression levels compared to those with lower expression levels within the same cell line. Meanwhile, to further investigate the relationship between RBP expression levels and the performance of HDRNet, we calculated their correlation. As depicted in Supplementary Fig. 9b, we found a weak positive correlation between the performance of HDRNet, as measured by metrics such as the AUC and RBP expression levels, suggesting that the binding sites of RBPs with higher expression levels were more likely to be accurately recognized by HDRNet.

Then, we analyzed the relationship between the performance of HDRNet and RBP expression levels from the perspective of dynamic prediction. As demonstrated in Supplementary Fig. 9c, we first computed the correlation of RBP expression levels in the K562 and HepG2 cell lines. We found a strong positive correlation between the RBP expression levels in these two cell lines, indicating similar RBP expression patterns between them. Therefore, we evaluated the dynamic prediction performance of HDRNet based on these categorizations. As depicted in Supplementary Fig. 9d, consistent with the static prediction results, HDRNet exhibited the most accurate dynamic predictive performance for these RBPs. However, it is worth noting that there are differentially expressed RBPs between the two cell lines. To identify these differentially expressed RBPs, we employed the DESeq2 package, as described in the reference (19). A total of 35 RBPs were identified, of which 15 were differentially expressed RBPs in K562 cells, and 20 were differentially expressed RBPs in HepG2 cells. Supplementary Fig. 9e visualizes the performance of HDRNet on these differentially expressed RBPs, confirming its superior performance in both cases. Based on these results, we further investigated whether the expression levels of RBPs influenced the dynamic prediction performance of HDRNet. As displayed in Supplementary Fig. 9f, we observed that the binding sites of RBPs with higher expression levels were more easily identified during the dynamic prediction process.

On the other hand, we conducted further analysis to evaluate the performance of HDRNet on RBPs with different target expression levels. RBPs play a crucial role in regulating gene expression by interacting with RNA and forming ribonucleoprotein complexes. However, the ENCODE project data (19) for RBPs in K562 and HepG2 cells does not provide expression values for their target RNAs, making it challenging to directly measure the high and low expression levels of target RNA events. Nevertheless, they have employed RNA sequencing after depleting RBPs using short hairpin RNA (shRNA) or CRISPR to investigate RBP target binding site functionality and examined expression level differences before and after knockdown (19). On this basis, there are 4 RBPs including DD3X3, DDX6, LARP4 and RBM15 that have previously been identified as RNA decay factors. After RBP-knockdown experiments, their target genes exhibited upregulation in specific cell conditions (knockdown-increased), indicating that these RBPs are associated with low expression levels of their target genes. Similarly, there are 6 RBPs including AKAP1, DDX55, APOBEC3C, FMR1, CPSF6, and IGF2BP3 that have been previously recognized to increase the stability of RNA targets; and their target genes showed downregulation after RBP knockdown in specific cell contexts (knockdown-decreased), indicating that these RBPs are associated with higher expression levels of their target genes. With these target RNA events with high and low expression levels, we first examined the static predictive performance of HDRNet on the specific cell line data for these RBPs. As depicted in Supplementary Fig. 10a,b, HDRNet consistently achieved the best performance and showed significant improvements compared to other baseline methods. For instance, in the RBM15_K562 dataset, HDRNet shows its performance gain of 8% over PrismNet; in the FMR1_K562 dataset, only HDRNet's AUC metric exceeded 0.9. These results indicate that HDRNet is capable of accommodating RBPs with different target expression levels and effectively identifying their binding characteristics. Furthermore, we selected those RBPs within this cohort that allowed for dynamic prediction and compared HDRNet's dynamic predictive performance. As demonstrated in Supplementary Fig. 10c,d, HDRNet also exhibited superior performance in dynamic prediction as well. For example, we observed a performance improvement of over 10% for RBM15 when comparing HDRNet to PrismNet. Notably, other baseline methods displayed considerable instability in the dynamic prediction tasks; for instance, DMSK failed to accurately identify the binding sites of DDX3X, and PrismNet performed poorly on AKAP1, with an AUC below 0.7. These results further validate the adaptability and robustness of HDRNet to RBPs with different target expression levels.

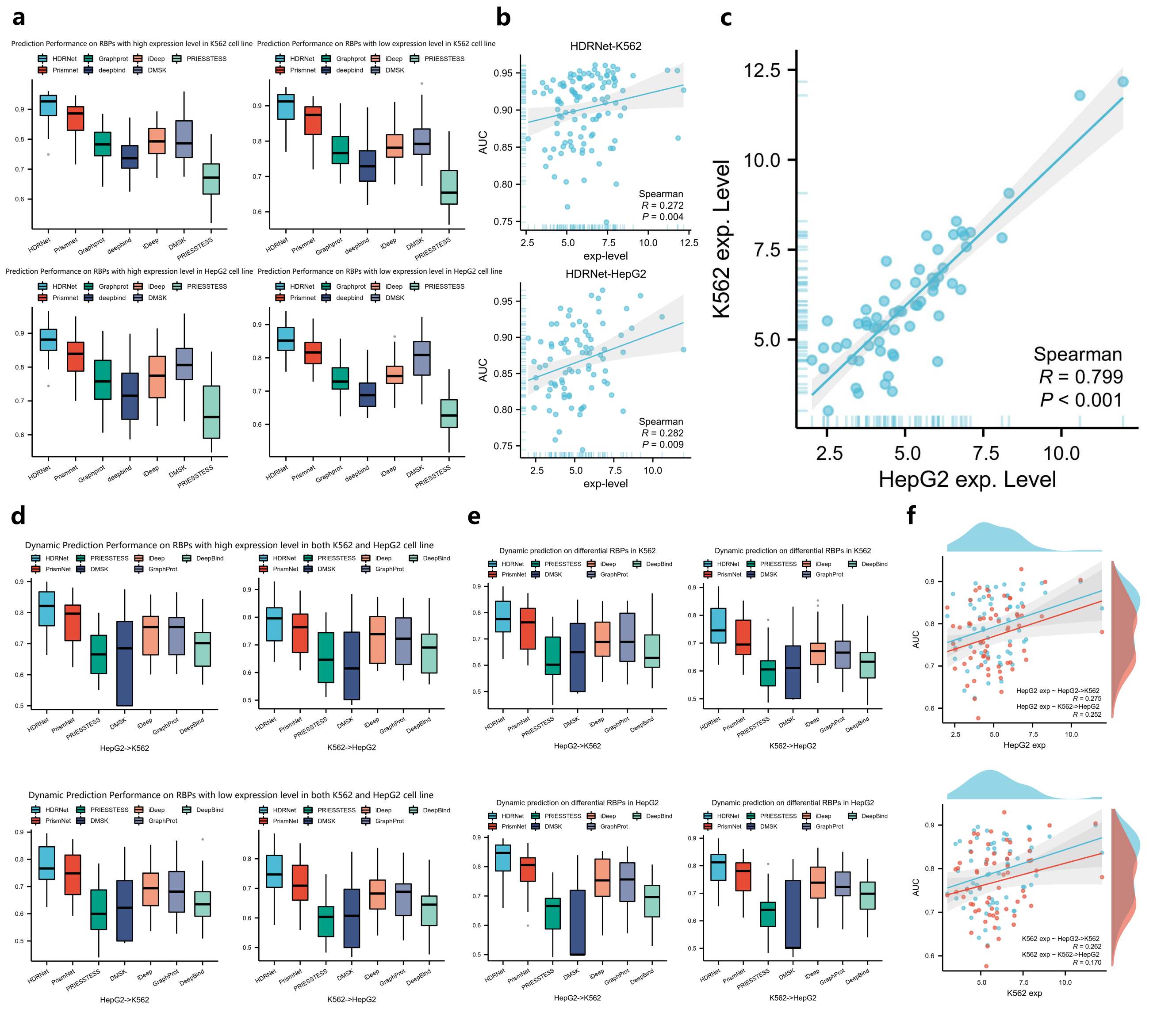

Supplementary Fig. 9. (a) Static prediction performance comparison of HDRNet and baseline models on different expression levels of RBPs (top: n=54 in each group; bottom: n=42 in each group; center line, median; box limits, upper and lower quartiles; whiskers, 1.5× interquartile range). (b) The correlation between HDRNet's performance and RBP expression levels. (c) The correlation of RBP expression levels between K562 and HepG2 cell lines. (d) The performance comparison of HDRNet on RBPs with identical expression levels across cell lines. (top: n=25 in each group; bottom: n=31 in each group; center line, median; box limits, upper and lower quartiles; whiskers, 1.5× interquartile range) (e) The performance comparison of HDRNet on differentially expressed RBPs across cell lines. (top: n=3 in each group; bottom: n=3 in each group; center line, median; box limits, upper and lower quartiles; whiskers, 1.5× interquartile range) (f) The correlation between RBP expression levels and HDRNet's dynamic prediction performance. Source data are provided as a Source Data file. (top: HepG2.exp HepG2->K562 p=0.0152, HepG2.exp K562->HepG2 p=0.0253; bottom: K562.exp HepG2->K562 p=0.0187, K562.exp K562->HepG2 p=0.0677; Pearson Correlation).

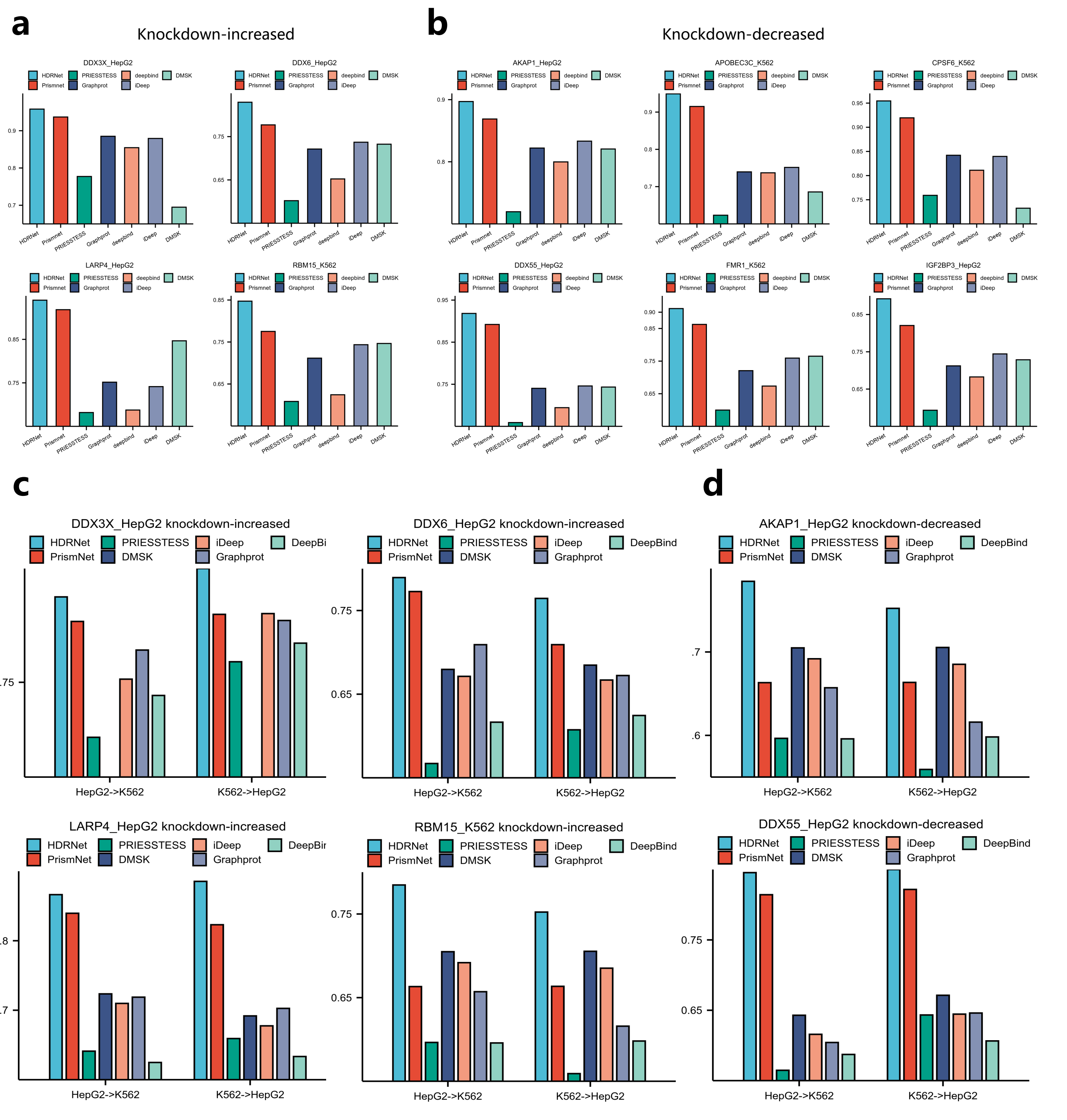

Supplementary Fig. 10. (a) The performance comparison of HDRNet on RBPs with lower target expression levels. (b) The performance comparison of HDRNet on RBPs with higher target expression levels. (c) The comparison of HDRNet's dynamic prediction performance on RBPs with lower target expression levels. (d) The comparison of HDRNet's dynamic prediction performance on RBPs with higher target expression levels. Source data are provided as a Source Data file.

Supplementary Note 8: HDRNet predicts dynamic RNA-RBP interactions on in vivo tissues under normal and disease conditions.

To demonstrate the effectiveness of HDRNet in both normal and disease conditions, we have meticulously curated the MBNL2 (Muscleblind Like Splicing Regulator 2) binding peak data (20) (GEO accession: GSE68890) in human brain tissues from POSTAR (21). This data source is comprised of five distinct datasets, including autopsy tissues (hippocampus and frontal cortex) from the patients with myotonic dystrophy type 1 (DM1, 2 datasets), myotonic dystrophy type 2 (DM2, 1 dataset of hippocampus), and control subjects (2 datasets), where DM1 and DM2 are progressive and multi-systemic neuromus-cular disorders, originating from the aberrant sequestration and activation of RNA processing factors and RAN translation. Then, we consolidated replicate data within each dataset and standardized the binding peaks to a length of 101 nucleotides. Subsequent evaluations of the dynamic prediction performance on these tissue datasets involved HDRNet, along with baseline models including PrismNet, DMSK, iDeep, GraphProt, DeepBind, and PRIESSTESS. Firstly, the evaluations focused on cross-tissue dynamic prediction experiments under single conditions, such as normal-normal and DM1-DM1 predictions. As shown in Fig. 4a, HDRNet consistently outperformed the other models across both conditions. Notably, we observed a significant performance gap between HDRNet and PrismNet in the cross-tissue prediction task, while PRIESSTESS failed to identify the binding sites of MBNL2 in the DM1 frontal cortex dataset, rendering it incapable of performing the dynamic prediction task. Then, to validate the efficacy of HDRNet in capturing dynamic tissue conditions between normal and diseases, we conducted experiments on cross-condition dynamic predictions, such as, we used the model trained on normal control tissue data to predict binding sites in disease tissues. As shown in Fig. 4b, HDRNet maintained strong dynamic prediction capabilities in cross-condition dynamic prediction. It achieved the highest AUC metric of 0.8, with an AUC difference exceeding 10% compared to PrismNet. These results demonstrate that HDRNet can effectively learn the binding patterns of RBPs across diverse conditions. Additionally, as highlighted in Fig. 4c and Supplementary Fig. 11a, HDRNet exhibited a robust capacity to detect and accurately capture disease-related high-attention binding regions. These regions represent the critical interaction sites between MBNL2 and DM1 extended CUG repeats and DM2 CCUG expansion RNAs, which are pathophysiological hallmarks of myotonic dystrophies, as previously revealed in (20, 22). By successfully capturing these regions, HDRNet not only aids in identifying key molecular interactions but also enhances our understanding of disease mechanisms.

To further investigate the predictive capabilities of our proposed model regarding dynamic interactions in tissues, we conducted an experiment to predict two additional eCLIP RBP datasets from adrenal tissues obtained from ENCODE, namely DGCR8 and HNRNPU. The experiment results are summarized in Supplementary Fig. 11b, where our model achieved the highest AUC metric. As depicted in this figure, we can observe that HDRNet achieved identical performance in dynamically predicting RBP binding in both K562 and HepG2 cell lines, while other baseline methods exhibited variability in performance when using models trained on different cell lines for dynamic prediction. This observation further highlights the ability of HDRNet to identify RBP binding patterns across different sources of RBP binding data. Interestingly, we also observed that PrismNet, although slightly inferior to HDRNet, showed improved performance on the new eCLIP data compared to the previous MBNL2 data. We speculate that this improvement is due to the fact that the PrismNet model was specifically designed based on eCLIP data. In particular, the static encoding within PrismNet limits its performance on other platforms. In addition, our observations revealed that the HDRNet model, trained on cell line data, successfully highlighted significant binding regions in the tissue binding data which were functionally relevant; for example, focusing on the DGCR8 protein, it has been reported to bind to extended CGG repeat sequences, leading to partial sequestration of DGCR8 in CGG RNA aggregates, consequently reducing the processing of miRNAs (23). In Supplementary Fig. 11c, we can observe that HDRNet effectively identifies the dynamic CGG repeat binding region, indicating its capability to dynamically identify RBP binding patterns. Additionally, HDRNet accurately identified the G-rich binding region of HNRNPU, as depicted in Fig. 4d. Notably, upon visualizing potential RNA models, we discovered that the identified G-rich binding regions predominantly corresponded to G-quadruplex structures, which are stable secondary structures formed by G-G base pairs. Indeed, as reported by (24), HNRNPU exhibits a preference for recognizing G-quadruplexes in RNAs as binding motifs. These results highlight the robustness of HDRNet on RBP data across different platforms, demonstrating that HDRNet with K562 and HepG2 cell line data is sufficient to accurately predict dynamic interactions in tissues.

Lastly, we have also added the direct regulatory targets of MBNL1 (Muscleblind Like Splicing Regulator 1) in brain, heart, muscle, and myoblasts from mice (Wang et al. Cell 2012, PMID: 22901804) (22) from the CLIP-Seq data to further explore the performance of HDRNet in different contexts. In particular, we collected a total of five datasets (GEO accession: GSE39911), including two from the brain (129Brain, B6Brain), one from muscle (B6Muscle), one from heart (B6Heart), and one from myoblasts (C2C12 cells), where 129 and B6 are individual mouse ID numbers. The experimental results, as illustrated in Supplementary Fig. 12a and Fig. 4e, demonstrate that HDRNet provided performance improvements compared to other baseline methods, resulting in an increase in AUC from 5% to 10%. Meanwhile, it is important to note that MBNL1 and MBNL2 are both members of the muscleblind-like (MBNL) protein family, and therefore MBNL1 shares similar binding patterns with MBNL2, as discussed previously, showing binding specificity to CUG and CCUG pathological expansions (20,

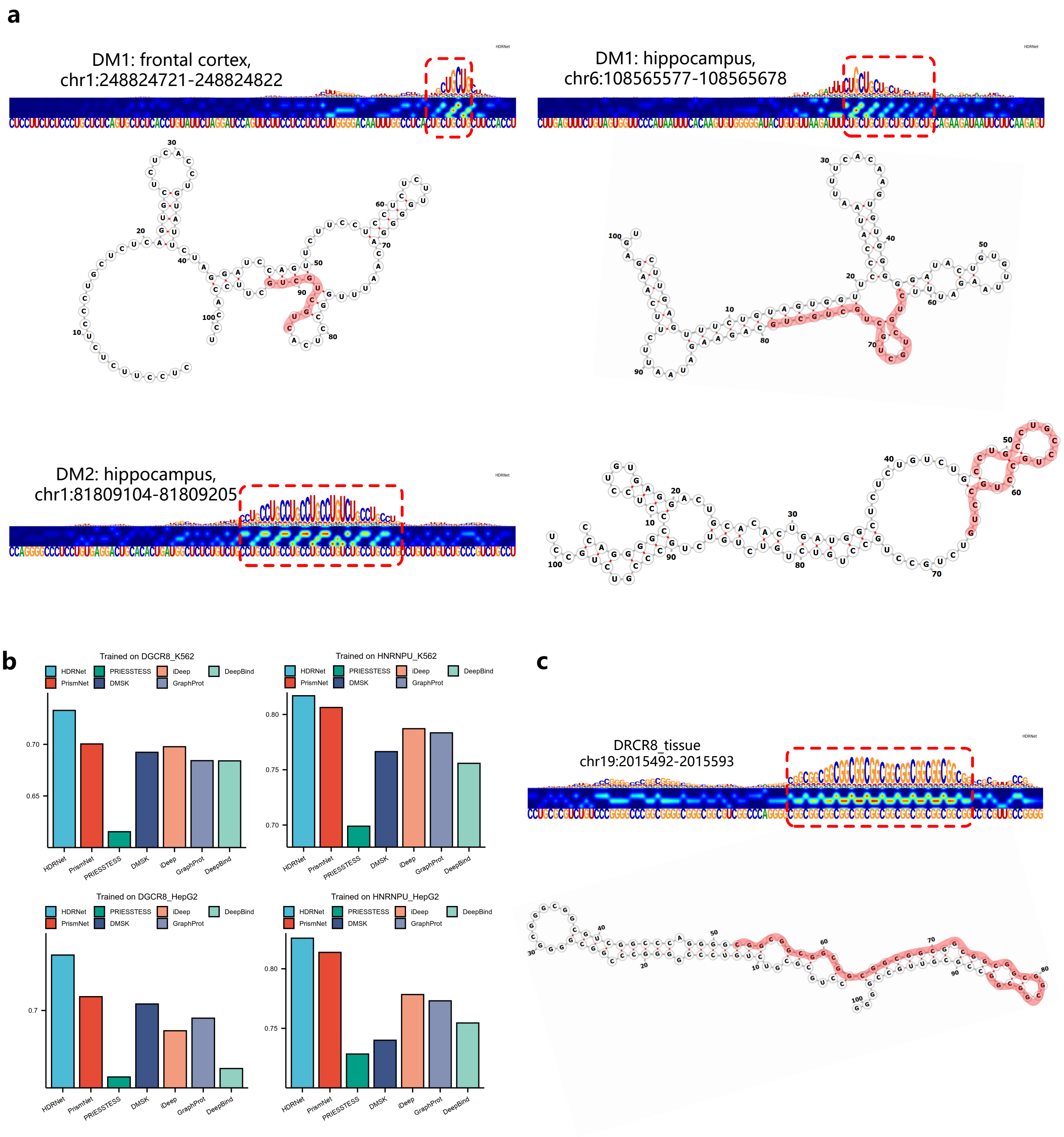

Supplementary Fig. 11. (a) The salient map of the high attention binding region captured by HDRNet. HDRNet successfully identifies the disease-related RNA repeats. (b) Performance comparison of DGCR8 and HNRNPU dynamic prediction, using the model trained on cell line data. (c) HDRNet identifies the CGG-rich region of DGCR8 binding patterns. Source data are provided as a Source Data file.

22). As illustrated in Supplementary Fig. 12b, HDRNet can dynamically distinguish these diseases-related RNA repeats sequences using models trained on different MBNL1 binding data, indicating that HDRNet can effectively extract RBP binding properties from the RBP binding data in different physiological environments. In summary, these results demonstrate the comprehensiveness of HDRNet in the task of dynamic prediction of RBP binding sites in multiple tissues.

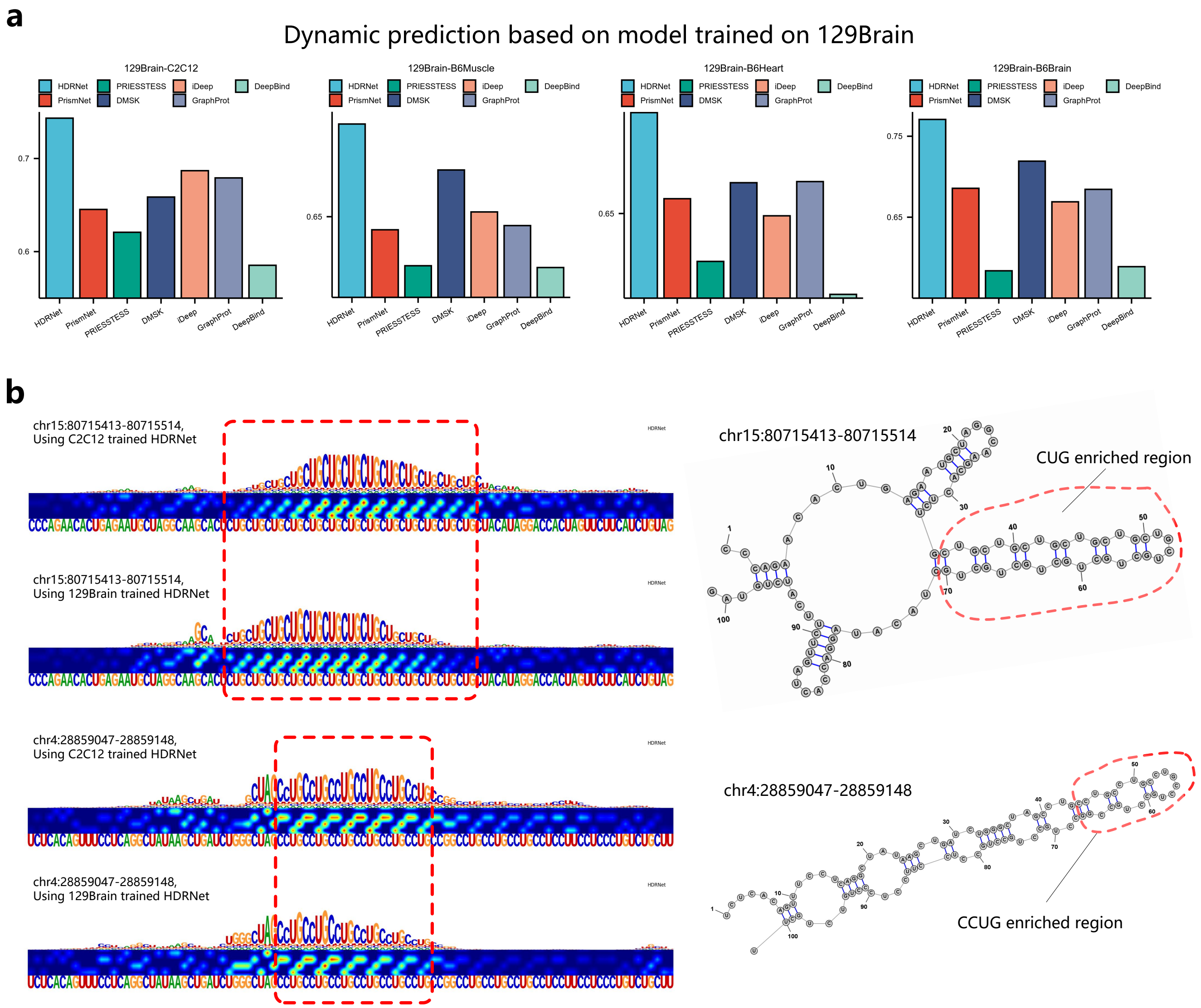

Supplementary Fig. 12. (a) Performance comparison of MBNL2 dynamic prediction performance using model trained on 129Brain date of mouse. (b) Salient binding regions identified by HDRNet under dynamic conditions. Source data are provided as a Source Data file.

Supplementary Note 9: Motifs identified by the dynamic global contextual embedding are highly comparable to known motifs.

Supplementary Table 1. Visualization of extracted motifs. We display 11 binding motifs matched in the ATtRACT database using the TOMTOM algorithm (two-sided Fisher-Irwin exact test and adopt Bonferroni correction to derive an E-value).

RBP	Known Motif	Extracted Motif	E-value	P-value
SRSF1	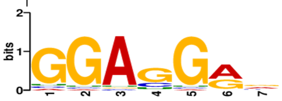	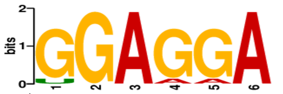	1.15e+00	7.23e-04
ELAVL	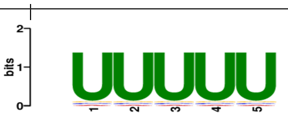	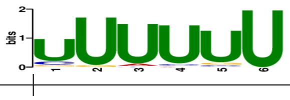	3.46e+00	2.18e-03
HNRNPA	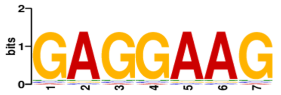	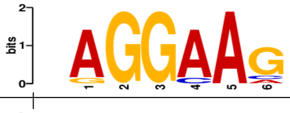	6.23e-01	3.94e-04
KHDRBS	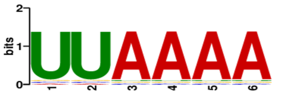	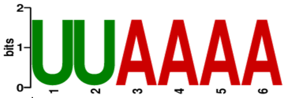	3.63e-01	2.30e-04
PABPC	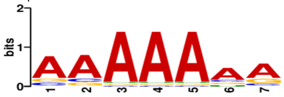	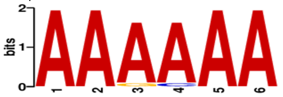	6.22e-01	3.93e-04
PCBP1	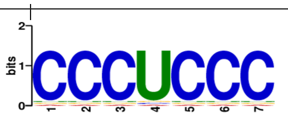	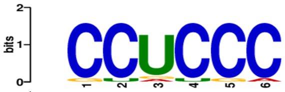	5.82e-02	3.68e-05
PCBP2	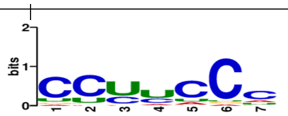	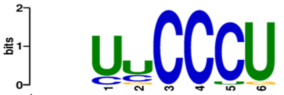	1.59e+00	1.00e-03
PTBP1	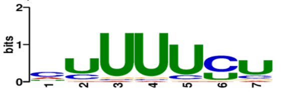	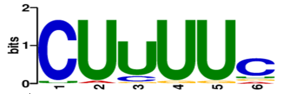	4.42e-01	2.79e-04
PUM2	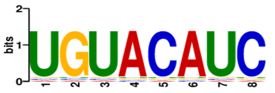	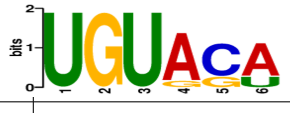	6.95e-01	4.39e-04
TIA1	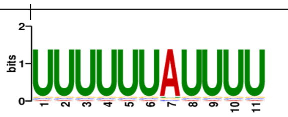	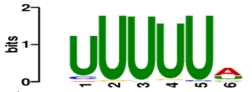	2.49e+00	1.57e-03
U2AF2	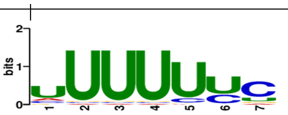	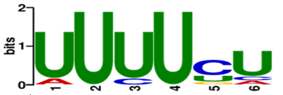	1.32e+00	8.33e-04

Supplementary Note 10: HDRNet captured the binding peaks associated with the extracted motifs and identified the structural preferences of the binding events.

We employed the SHAP tool (25) to extract high-attention dynamic semantic information. We found unexpectedly that HDRNet captured the binding peaks associated with the extracted motifs and identified the structural preferences of the binding events. Taking the input sequence of RBP TIA1 in Hela cells as an example, Supplementary Fig. 13a reflects the impact of each token of the input dynamic contextual features and the icSHAPE structural information, where the darker color indicates the positive effect portion of the input sequence. The darker the color of the sequence, the more attention HDRNet devotes to that part of the sequence. We can see that HDRNET identified the binding peaks of the sequence and the secondary structure at the same position, where the tokens are UUU, UUU, UUU, UUA, which correspond to the poly-U binding motifs we identified using transformers (UUUUU). Moreover, the binding peaks of secondary structures revealed the structural preference of RBP-RNA binding behavior (single-stranded: icSHAPE score>0.8, double-stranded: icSHAPE score < 0.2), as plotted in Supplementary Fig. 13b. From this figure, the high-attention binding peak appearing at position 40 is the single-stranded poly-U (UUUUU) fragment of the TIA1 binding site corresponding to the extracted binding peak, which is in line with the confirmations of earlier investigations (18). In addition, we compared the identified motif with known motifs from the ATtRACT database and found it to be highly compatible as shown in Supplementary Fig. 13c, corroborating the biological interpretability of HDRNet.

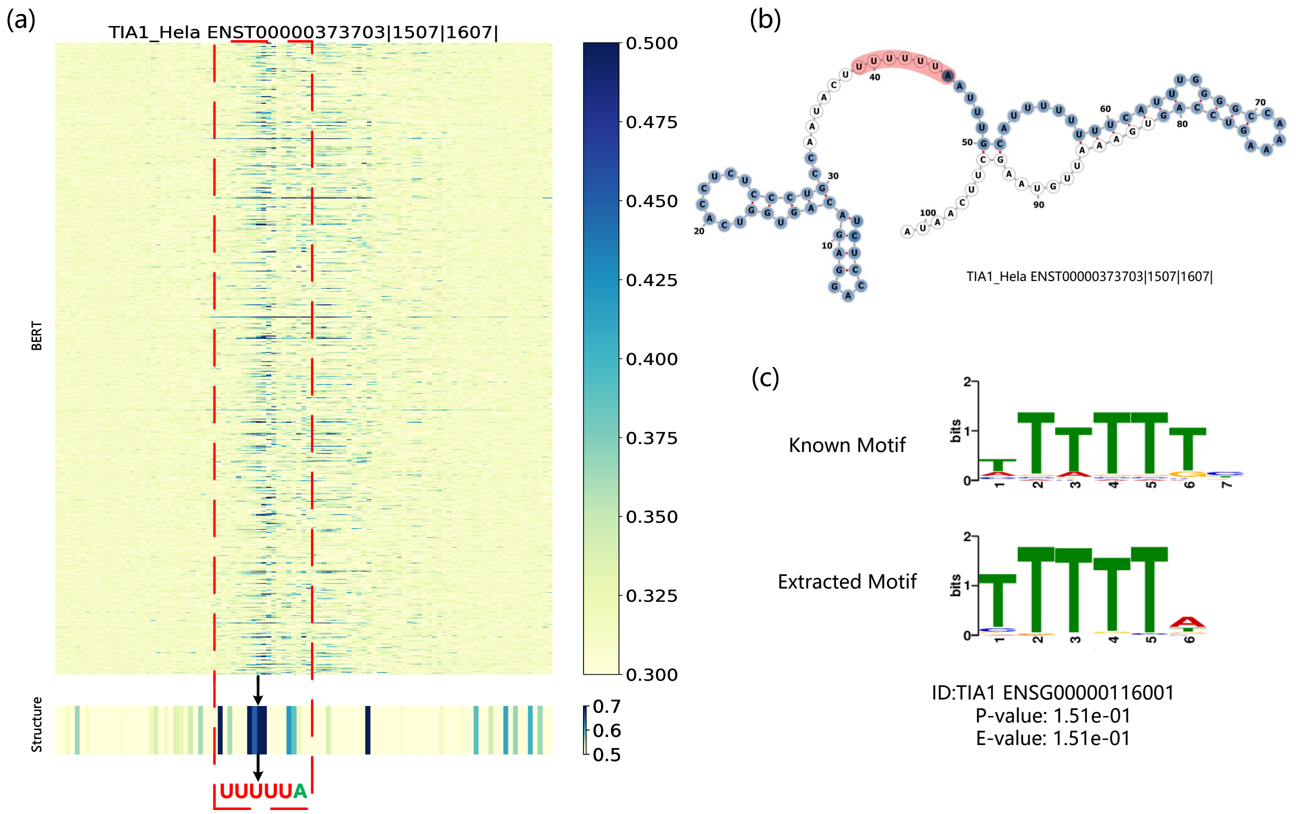

Supplementary Fig. 13. HDRNet successfully capture the high-attention Poly-U binding motif 'UUUUU' with single-stranded structural binding preference. (a) The high-attention binding peaks of sequence and structure are focused on specific tokens. (b) Visualization of the RNA structure, which is consistent with the identified motif secondary structure. (c) Comparison of the identified binding motif with the known motif from ATtRACT database.

Supplementary Note 11: Visualization of the self-attention mechanism of the BERT model

We evaluated the ability of HDRNet to extract dynamic contextual information from BERT, by exploring the distribution of attention weights in BERT from HDRNet. The top half of Supplementary Fig. 14 illustrates all the attention heads in the third (shown in green) and fourth layers (shown in red), corresponding to the given sequence, and demonstrate that the attention scores of each head accumulate in the key fragments of the sequence that have a large impact on the identification decision; the bottom half of Supplementary Fig. 14 shows the attention calculation process corresponding to the input sequence, where the attention scores between different tokens can be calculated based on the *Query q* vector and *Key k* vector, as described in the Section "Dynamic Global Contextual Information". In Supplementary Fig. 14, the positive values are displayed in blue and the negative values are in orange. Using the token "TTT" in the input sequence as an illustration, the attention distribution from the first attention head of the seventh layer is visualized, from which we observe that the attention values between token "TTT" and other tokens in the selected attention head do not decay noticeably with increasing distance, indicating that BERT successfully learned and preserved the long-distance dependencies and short-distance context of the sequence.

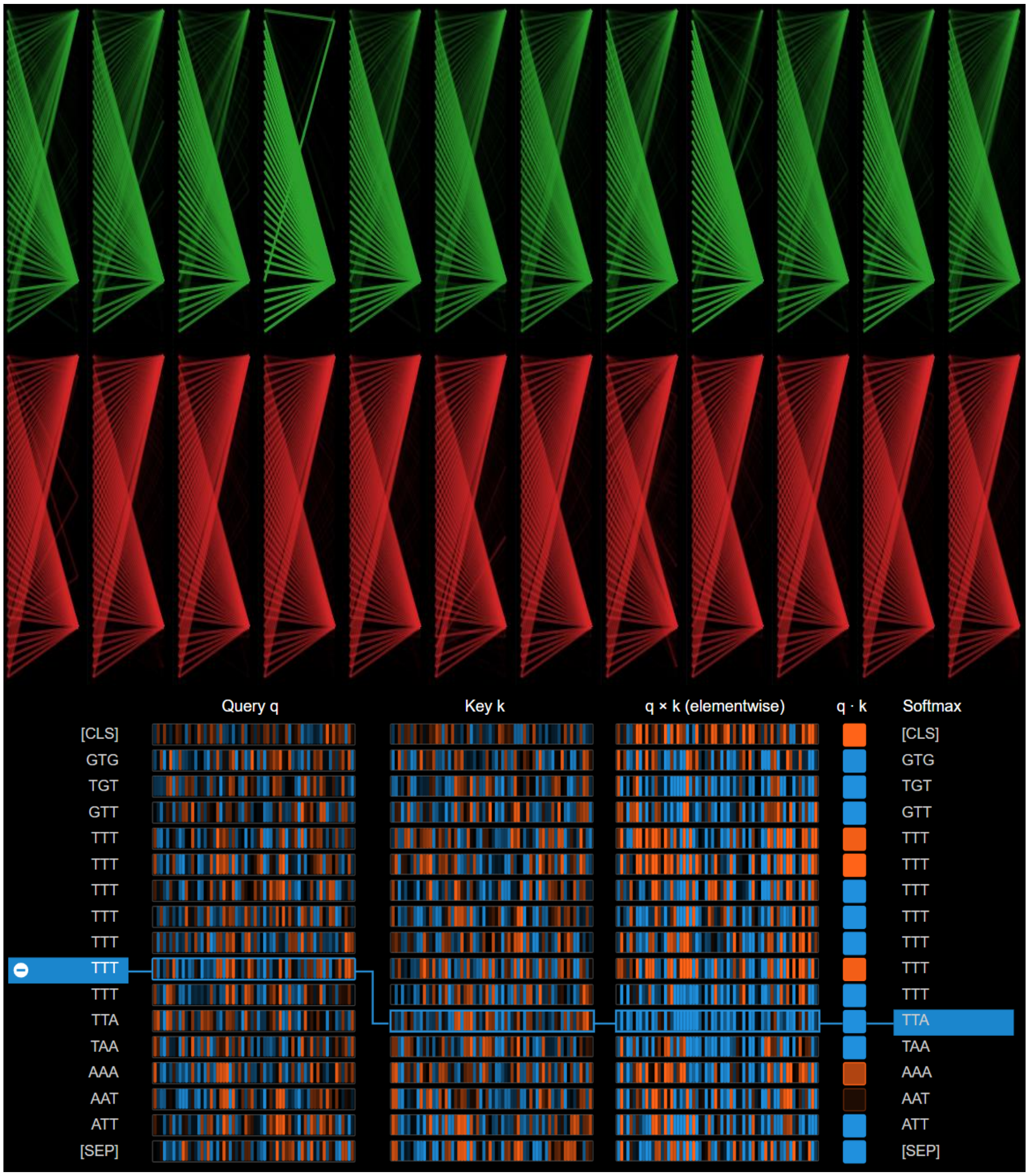

Supplementary Fig. 14. Top half shows a bird's eye view of the attention distribution of the different attention heads in two different layers of BERT, where the rows represent different layers of BERT consisting of 12 attention heads in the Transformers layer. The attention scores of each attention head gradually concentrate in the key regions of the input RNA sequence; the bottom half visualizes the process of attention score calculation, where the first and second columns represent the query vector and key vector, respectively. The framed up vectors show the two most relevant tokens in the RNA sequence.

Supplementary Note 12: HDRNet can detect high-attention binding region with variants allels.

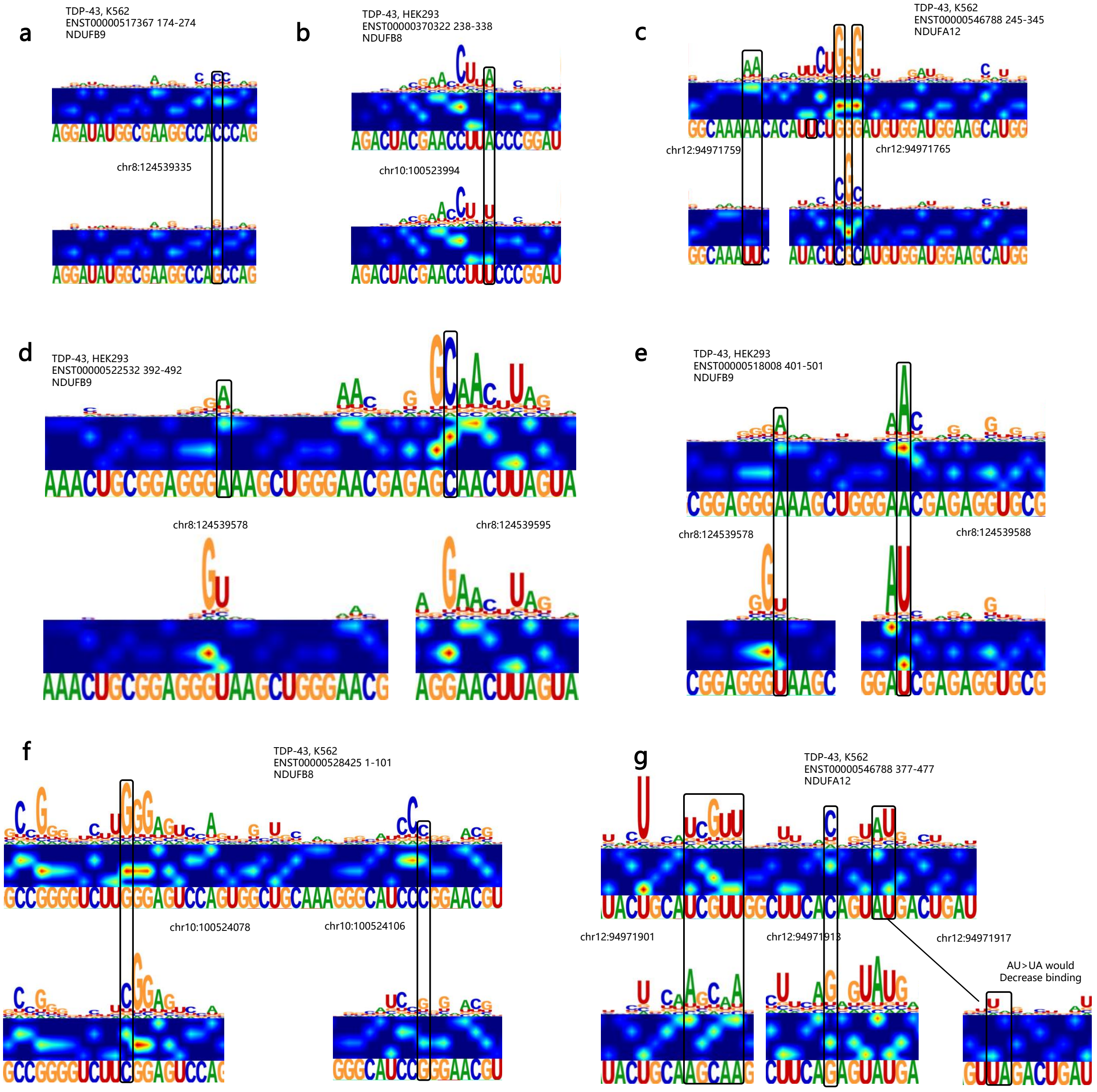

Supplementary Fig. 15. The high-attention binding regions captured by HDRNet are enriched with genomic variants. (a) 5 prime UTR variant that is linked with Large intestine tumour. (b) A URT variant of the 3' UTR. (c) SNP variants that changes non-coding exon sequence in a non-coding transcript. (d) Transcripts variants occurring within an intron. (e) SNP variants located in the intron region. (f) A 3 prime UTR variant and a coding sequence SNV variant that is possible lead to Skin tumour. (f) Non-coding transcript exon variants. (g) The SNV variant located in chr12:94971913 is possible related with Biliary tract tumour and Stomach tumour.

Supplementary Note 13: Nucleotide mutations lead to potential RNA structural changes.

As depicted in Supplementary Fig. 16, we visualized RNA models with significant structural alterations. In particular, we employed RNAfold to predict and visualize the possible RNA structure after locus mutation, and then compared it with the predicted secondary structures constrained by icSHAPE. Supplementary Fig. 16a summarizes the high-attention binding regions of the NDUFA12-binding transcript variant (ENST00000547986) of TDP-43 identified by HDRNet in K562 cells, both before and after mutations, along with the corresponding predicted RNA secondary structure models. From this figure, we can observe that the mutations occurring at positions 94971518 and 94971529 on chromosome 12 could potentially lead to considerable change in the overall structure of the segment after the local variation; for instance, an A->U mutation at position 25 of the sequence results in a loss of pairing with U at position 61, instead pairing with A at a closer position, i.e. 47. Additionally, the AUUUUU fragment at positions 37 to 42 forms an expanded loop structure post-mutation.

Similar structural changes were also identified in transcript ENST00000517367, the NDUFB9 binding gene of TDP-43 in HEK293 cells. As demonstrated in Supplementary Fig. 16b, the mutations at positions 124539290 and 124539313 on chromosome 8 also lead to structural variants, where the C->G mutation at position 2 and the G->C mutation at position 25 result in pairings that would not ordinarily occur in that segment: the G at position 2 paired with the C at position 100, and the C at position 25 paired with the G at position 10. Furthermore, Supplementary Fig. 17 provides the influence of RNA base mutations on global structural changes. As depicted in Supplementary Fig. 17a, the G->C mutation at position 40 within the ENST00000276689 transcript does not modify the pairing behavior at that particular position; however it leads to an significant alteration in the overall structure of the segment. Similarly, Supplementary Fig. 17b highlights the impact of the AA->UU mutation at positions 47-48 in ENST00000517367, inducing a structural shift. For instance, the C at position 10 no longer pairs with the G at position 23 but instead aligns with the G at position 70, while preserving its pairing behavior at the mutation site. Overall, the above analysis illustrates that sequence variants may lead to substantial structural alterations within RNA sequences, which in turn affects the binding behavior.

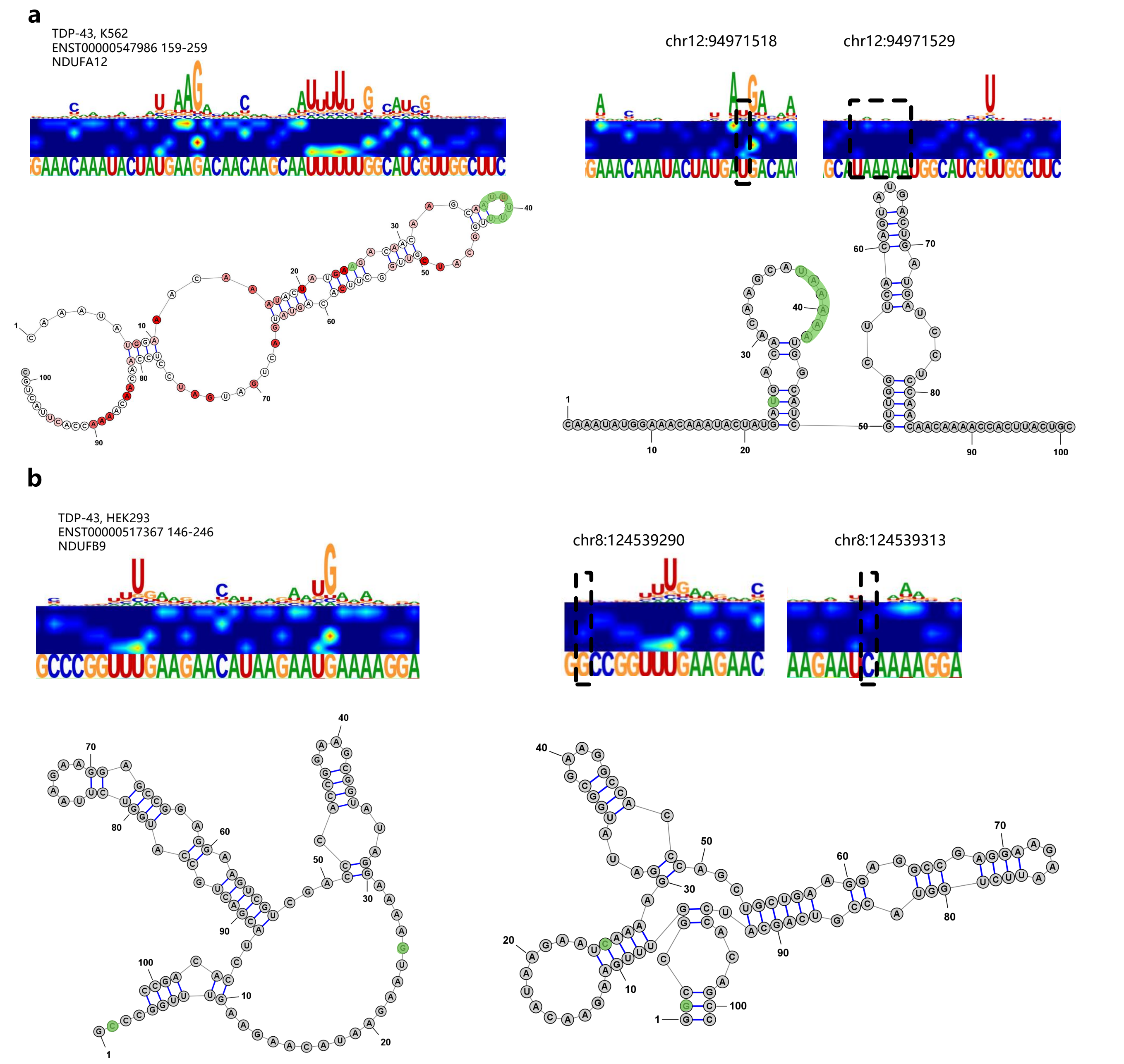

Supplementary Fig. 16. Nucleotides mutation can potentially lead to local structural variations in RNA. (a) The mutation led to the formation of two branches in the original stem-loop structure. (b) The mutation altered the local stem-loop structure arrangement.

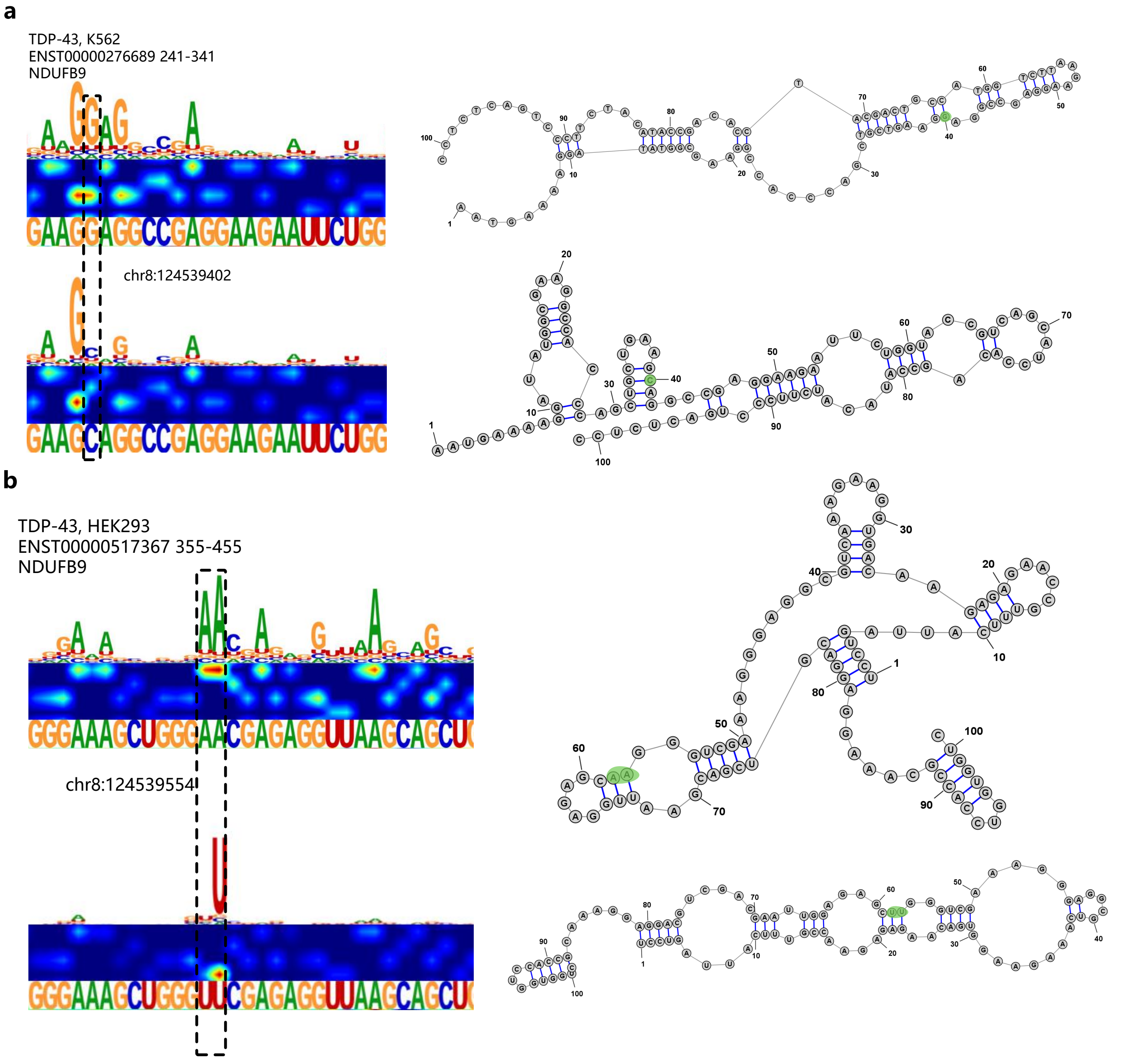

Supplementary Fig. 17. Alterations in nucleotides can potentially induce variations in global RNA structure. (a) A single nucleotide mutation resulted in changes in the overall arrangement of the RNA stem-loop structure. (b) The mutation resulted in the transformation of a multi-branched loop structure into a single-branched stem-loop structure.

Supplementary Note 14: Protein-Protein interaction (PPI) analysis of the identified dynamic binding genes from TDP-43 binding transcripts

After mapping the TDP-43 binding transcripts into gene symbols, we fed the TARDBP binding genes into the STRING (26) to build a protein-protein interaction (PPI) network to visualize the interactions and adhesion pathway. As depicted in Supplementary Fig. 18, a total of 537 nodes and 1851 edges were obtained, where neurological disease-related genes are highlighted (Parkinson's disease - red, Amyotrophic lateral sclerosis - blue, Huntington's Disease - green, Prion Disease - yellow, Alzheimer's disease - pink), and we observe that these genes are highly correlated and clustered. We then further extracted all nodes that are related with diseases (a total of 68 nodes) and observed their interactions. As shown in Supplementary Fig. 19, we noticed that genes with similar regulatory functions were grouped together, suggesting a potential association of dynamic regulation across diverse cellular conditions with disease.

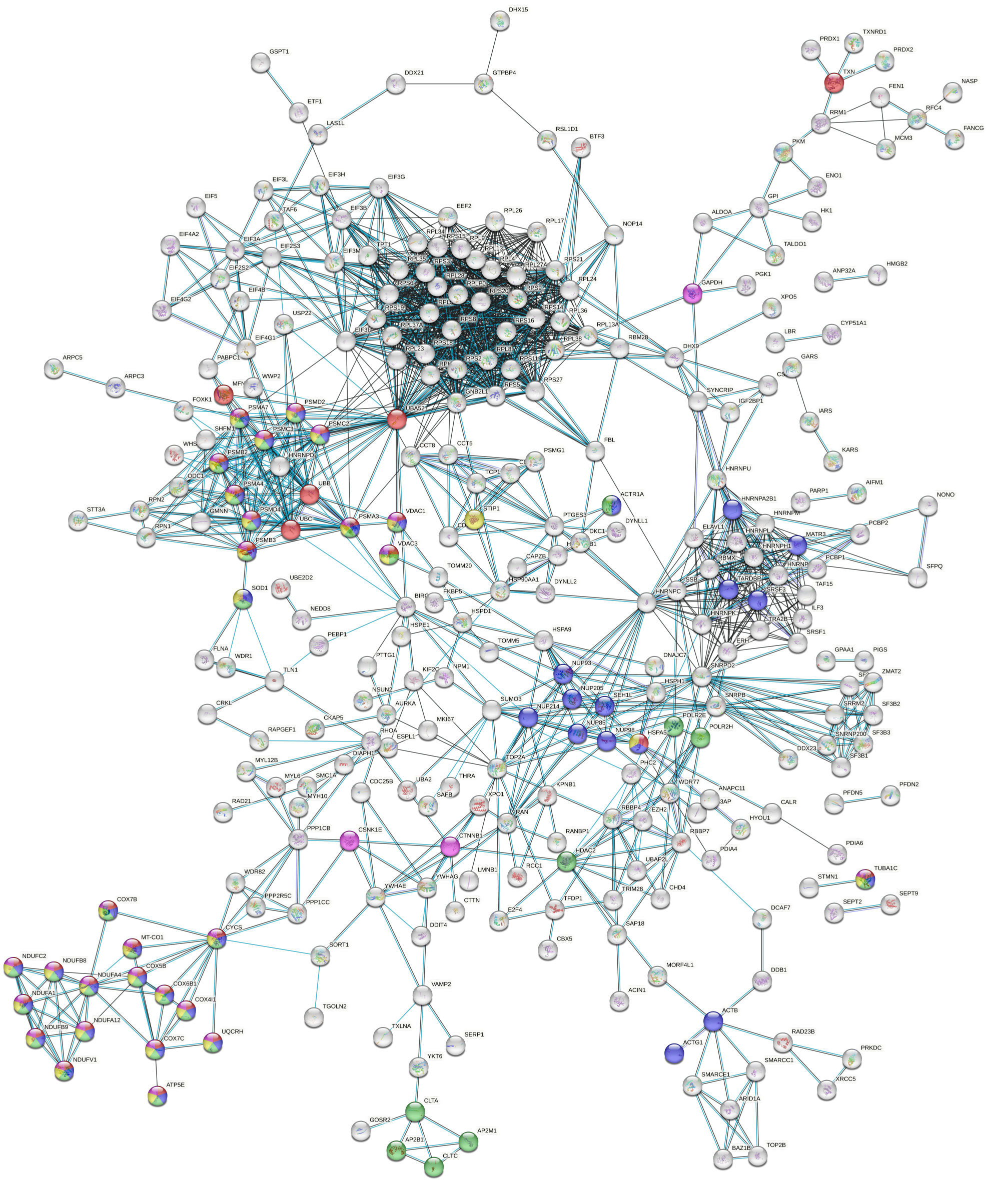

Supplementary Fig. 18. The protein-protein interaction network of the shared TARDBP binding genes between K562 cells and HEK293 cells for neurological diseases localization. The entire network consists of 537 nodes and 1851 edges. Only the protein markers with the highest confidence of interaction score are shown here. In particular, all neurological disorder-related markers (a total of 68 genes) are colored for easy observation.

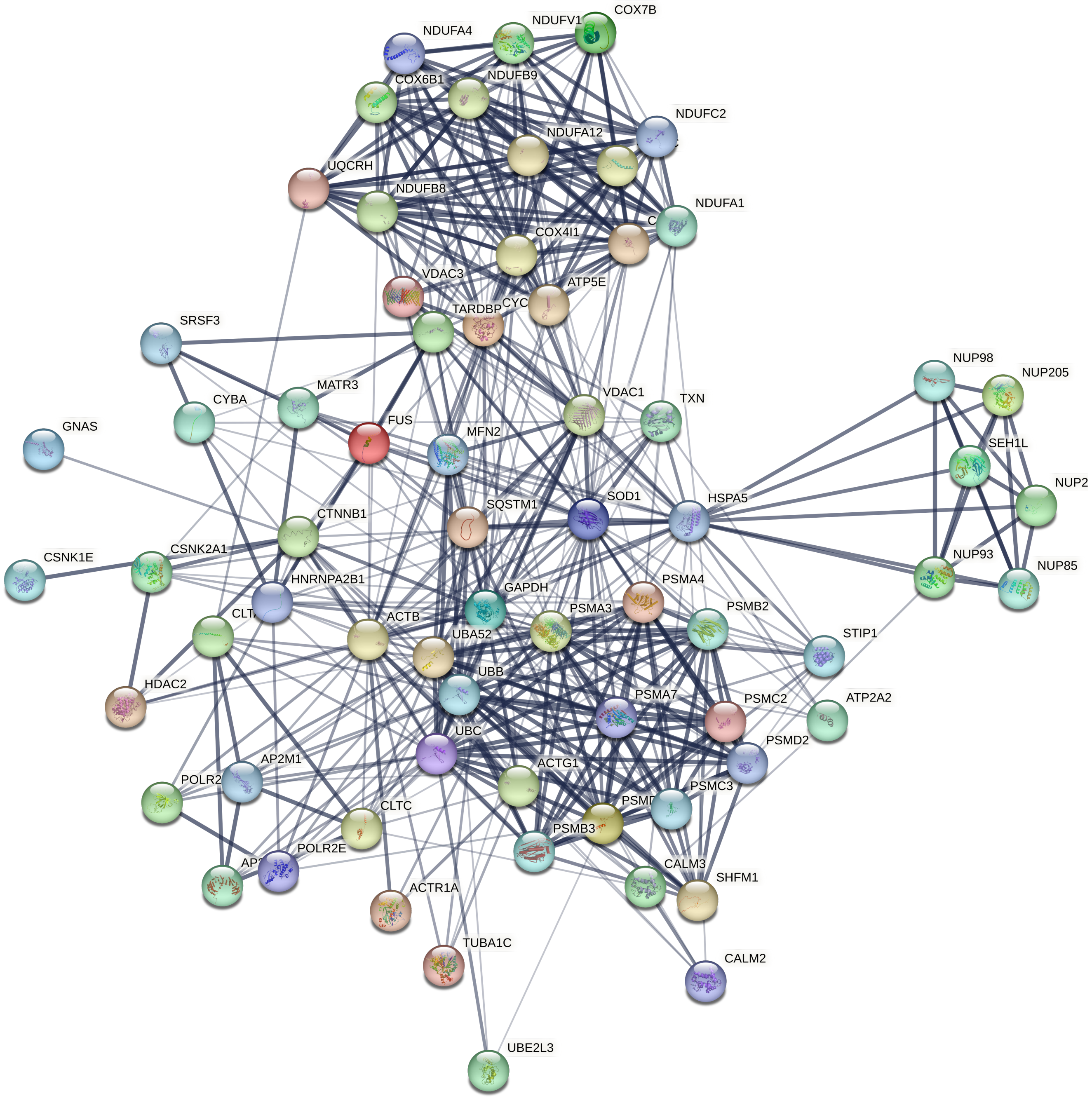

Supplementary Fig. 19. The protein-protein interaction network of the 68 diseases related genes, where genes with similar regulatory functions were grouped together.

Supplementary Note 15: Pan-cancer analysis under the identified hub-genes

Focusing on the top hub TDP-43 binding genes, we performed the pan-cancer analysis to characterize the differential expression of these genes in various cancers using TIMER (27). As depicted in Supplementary Fig. 20, we observed that these genes are also significantly differentially expressed between tumors and normal tissues in human cancers. Notably, some of the cancers identified (Breast Cancer (BRCA), Lung Squamous Cell Carcinoma (LUSC), Lung Adenocarcinoma (LUAD) and Colon adenocarcinoma (COAD) were consistent with those we found in RBP binding site mutations, thus corroborating the potential association of RBPs with cancer.

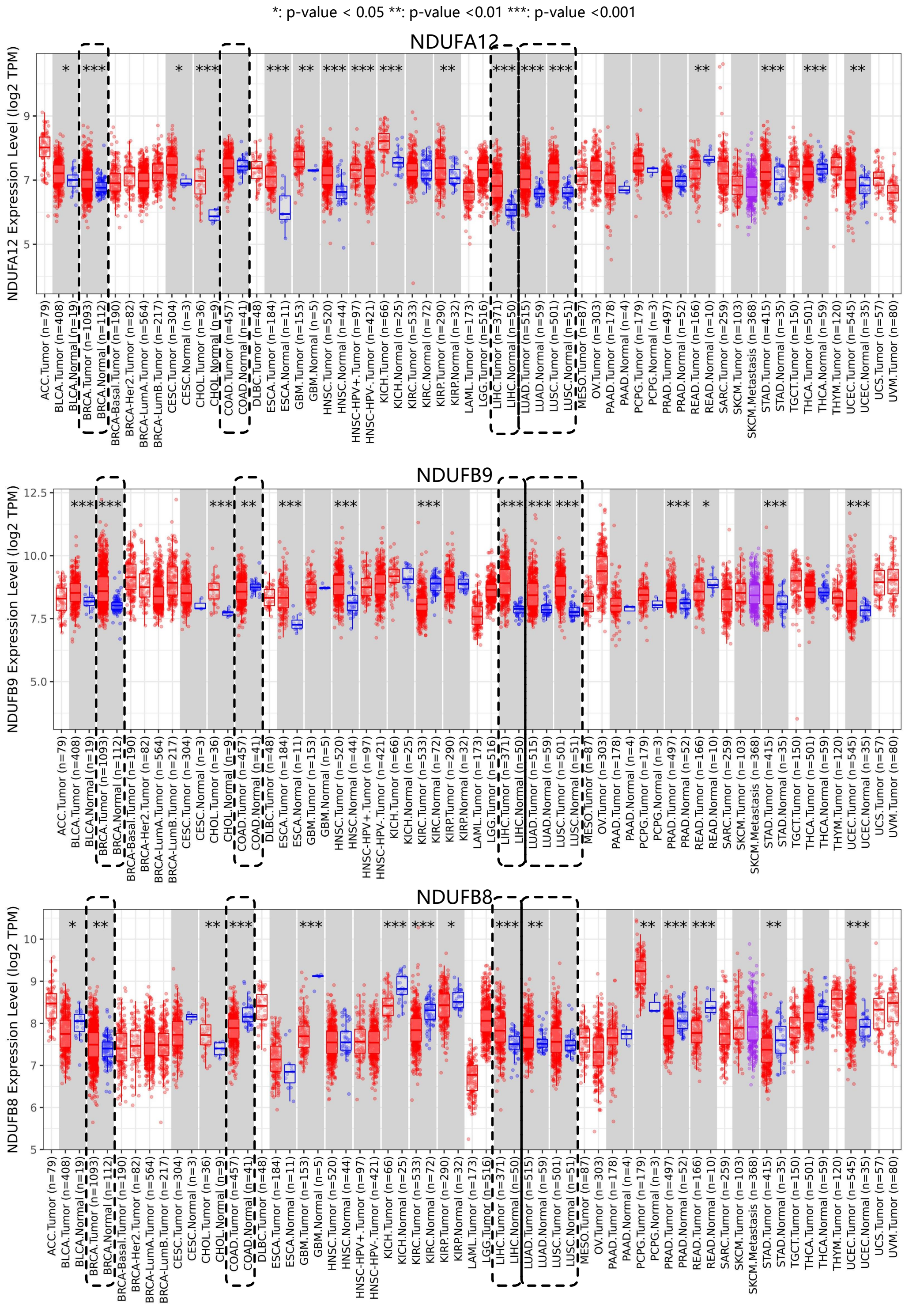

Supplementary Fig. 20. The differential expression of three hub genes between tumors and adjacent normal tissues in the TCGA pan-cancer dataset. Significance is determined by two-sided Wilcoxon test (adopt BH to adjust p-values for multiple comparisons, and the exact p-values for NDUFA12: BRCA, 3.52E-12 ; COAD, 0.178340797 ; LIHC, 5.57E-23 ; LUAD, 1.22E-14 ; LUSC, 1.39E-21 ; NDUFB9: BRCA, 8.87E-31 ; COAD, 0.005764774 ; LIHC, 5.81E-24 ; LUAD, 5.31E-13 ; LUSC, 2.46E-25 ; NDUFB8: BRCA, 0.00808 ; COAD, 7.19E-10 ; LIHC, 7.24E-08 ; LUAD, 0.003843805 ; LUSC, 0.06832645) Source data are provided as a Source Data file.

Supplementary Note 16: Hub gene-disease association network

We explored the hub gene-disease association using NetworkAnalyst (28), and the gene-disorder association network is depicted in Supplementary Fig. 21. Among the related diseases, we noticed that *NDUFB9* is apparently linked with liver disease. Meanwhile, from the previous pan-cancer analysis, we also found that NDUFB9, NDUFB8 and NDUFA12 were all significantly associated with liver cancer. We noticed that NDUFB9, NDUFB8 and NDUFA12 protein are all subunits of NADH dehydrogenase, suggesting that the NADH dehydrogenase may have an essential association with liver tumors.

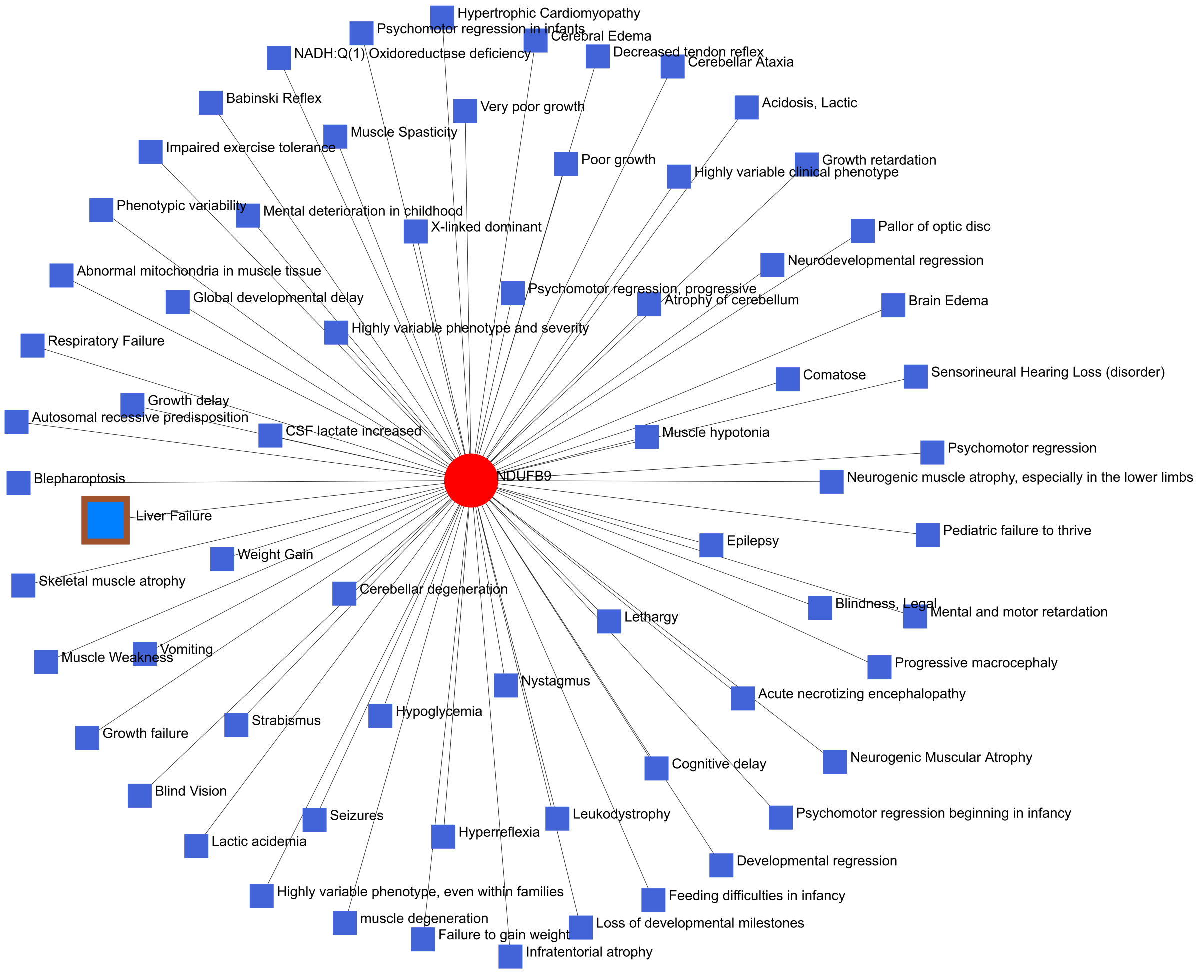

Supplementary Fig. 21. The gene-disease association network represents diseases associated with the hub TDP-43 binding genes. The disorders are depicted by the blue square nodes and the subsequent gene symbol is defined as the red circle node.

Supplementary Note 17: Interaction network of disease-associated genes with TFs and miRNAs

We focused on the 68 disease-related genes that are linked to neurological diseases and looked at their interactions with TFs and miRNAs to find transcriptional and post-transcriptional regulators of disease-related TARDBP-binding genes. From the perspective of TF-gene interactions, as illustrated in Supplementary Fig. 22, the identified TFs, FOXC1, GATA2, YY1, NFIC, PPARG, E2F1, NFYA, SRF and RELA are associated with different types of neurological diseases. Further, as visualized in Supplementary Fig. 23, some of the identified miRNAs are involved in neurological disorders (e.g., hsa-mir-16-5p (29), hsa-mir-92a-3p (30), hsa-mir-615-3p (31), hsa-mir-93-5p, (32), hsa-mir-320a (33), hsa-mir-484 (34), hsa-mir-106b-5p (35), hsa-mir-125b-5p (36), hsa-mir-26b-5p (37), hsa-mir-15b-5p (38). Indeed, we see that most of the miRNAs were linked to different kinds of human diseases, especially diseases relating to the nervous system.

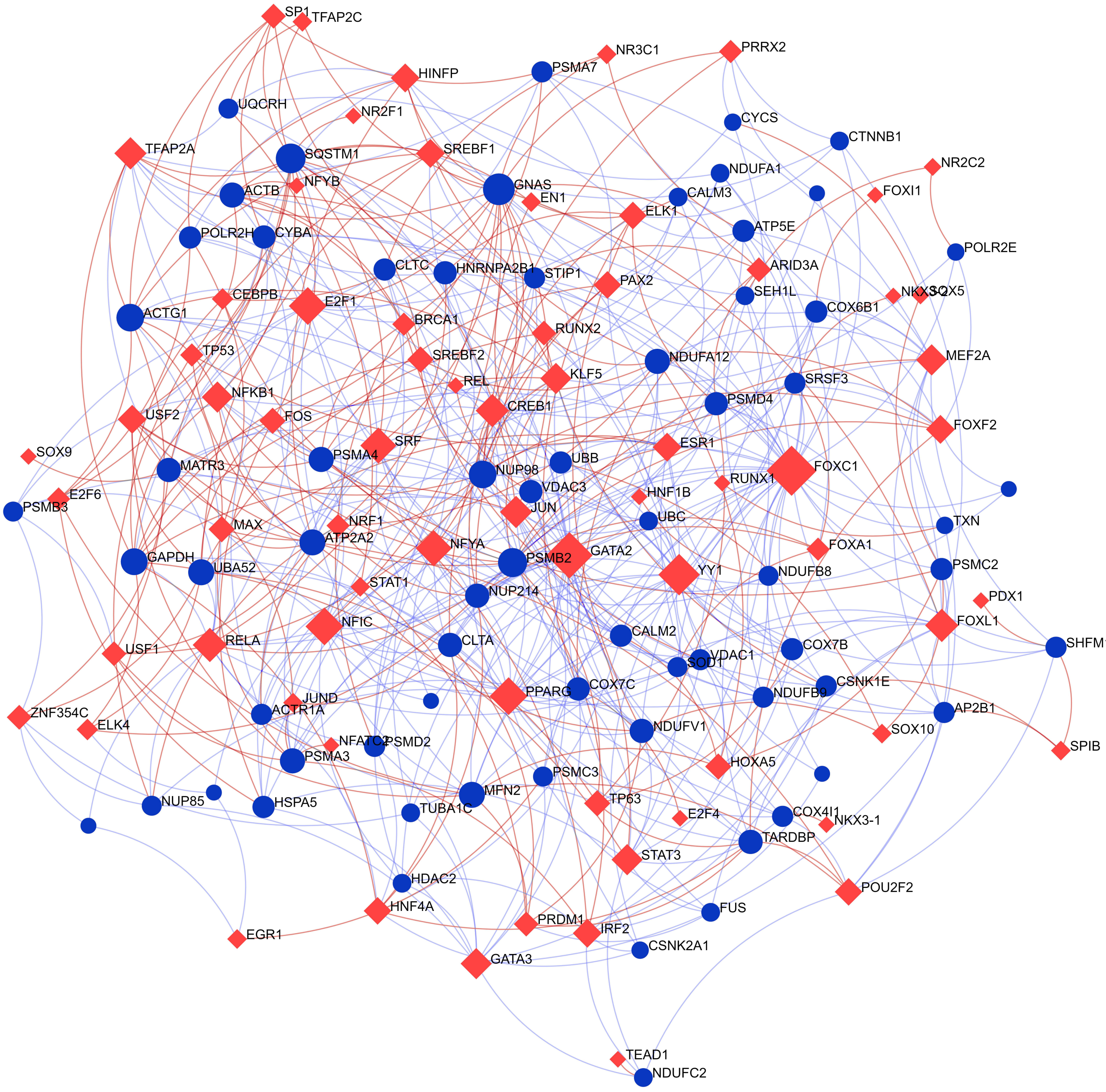

Supplementary Fig. 22. The cohesive regulatory interaction network of genes–TFs obtained from the Network Analyst, where the diamond nodes are gene symbols, and TFs interact with the genes as circle nodes.

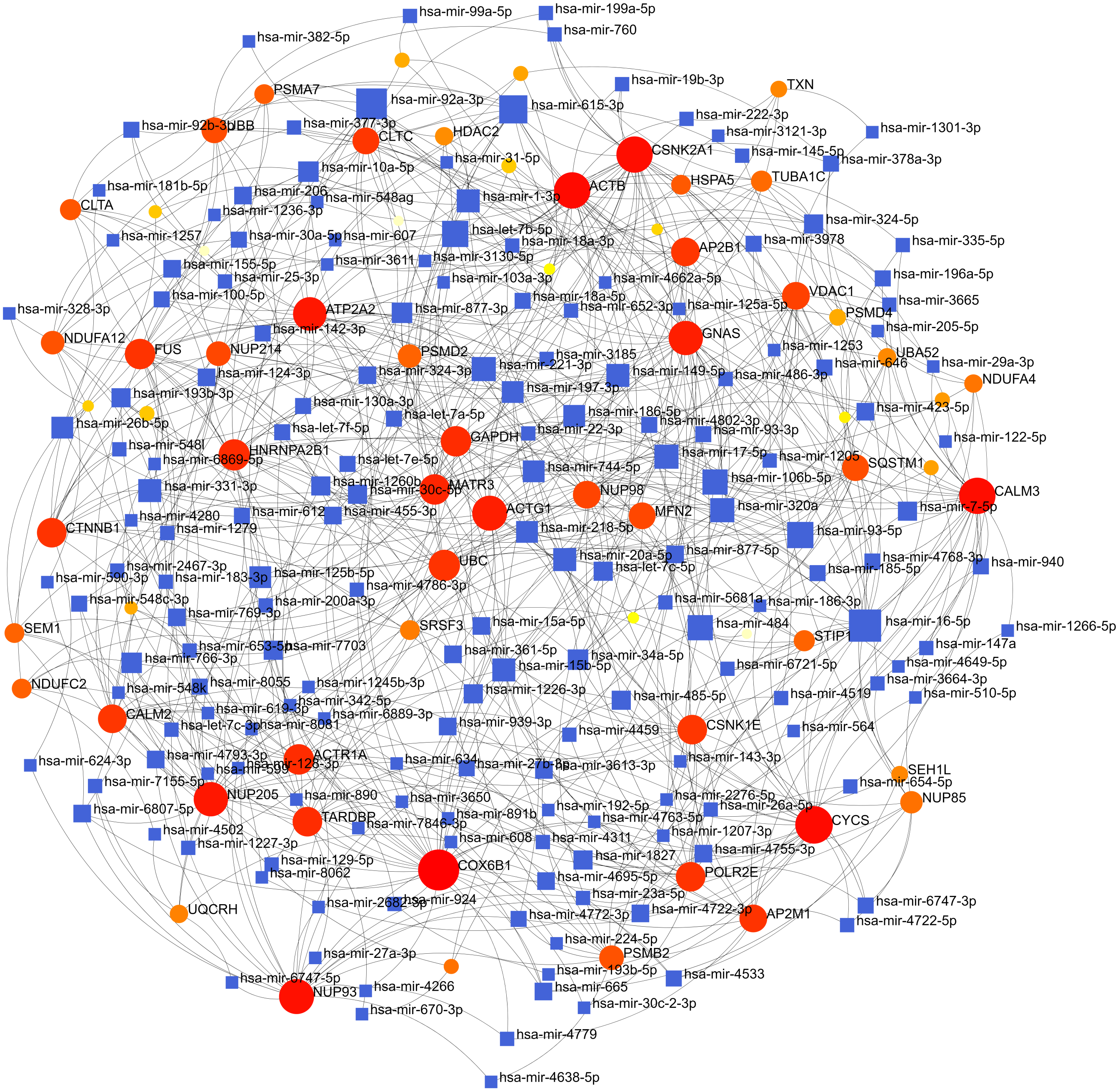

Supplementary Fig. 23. The interconnected regulatory interaction network of genes–miRNAs, where the blue square nodes indicate miRNAs and red circle nodes the genes interacting with the miRNAs.

Supplementary Note 18: Possible drug molecules for treatment of neurological disorders

Considering all 68 identified genes associated with neurological diseases as potential drug targets, we employed Enrichr to access the Drug Signatures Database (DSigDB) and conducted an analysis to identify potential drug molecules for the treatment of neurological diseases, using transcriptomic signatures. As shown in Supplementary Table 2, we extracted the top 10 chemical compounds that could be potential candidates for neurological diseases therapies based on the p-Value< 0.001; for instance, we identified ambroxol, a mucolytic compound predominantly prescribed for respiratory conditions like chronic bronchitis (39). Interestingly, recent studies have uncovered ambroxol's role as a pharmacological chaperone for glucosylceramidase (GCase), an enzyme that is often found to have reduced activity in the brains of Parkinson's disease patients, potentially contributing to the degeneration of dopaminergic neurons (40). Given ambroxol's ability to augment GCase levels, it holds promise as a potential disease-modifying intervention for Parkinson's disease (41–43). Moreover, ambroxol is also a candidate drug for Amyotrophic Lateral Sclerosis as it promotes and protects motor units and improves axonal plasticity (44).

We also extracted Metformin hydrochloride, a well-known antidiabetic medication commonly used to manage type 2 diabetes. However, emerging studies have suggested that metformin may have a role in the regulation of neurological systems (45). Indeed, research has indicated that Metformin has the potential to delay the progression of Alzheimer's disease by improving insulin sensitivity and reducing inflammatory responses (46, 47). Moreover, evidence suggests that metformin could provide neuroprotection by activating the AMP-activated protein kinase (AMPK) pathway, which could help alleviate symptoms of Parkinson's disease, as in Katila et al. (48). Another compound we extracted was Carfilzomib, a specific proteasome inhibitor primarily used for the treatment of multiple myeloma, particularly in cases that are refractory to other treatment modalities or have relapsed (49). Extensive research has been conducted on the relationship between the proteasome and neurodegenerative diseases, such as Alzheimer's disease, Parkinson's disease, and Huntington's disease (50–52). Under these conditions, the abnormal accumulation of specific proteins could lead to neuronal damage and death (52, 53). Intriguingly, Carfilzomib functions as an epidermal growth factor receptor (EGFR) inhibitor, which has been reported to be associated with susceptibility to Parkinson's disease (54, 55).

In conclusion, our candidate drug prediction analysis identified several promising drugs with potential value for the treatment of neurological disorders. These findings warrant further investigation and provide possible insights into novel therapeutic strategies for those complex conditions.

Supplementary Table 2. **List of the candidate drugs for neurological diseases (two-sided Fisher-Irwin exact test). Source data are provided as a Source Data file.**

ID	Name	P-Value	Chemical Formula	Chemical structure
1	clindamycin HL60 DOWN	1.98791E-12	$C_{18}H_{33}ClN_2O_5S$	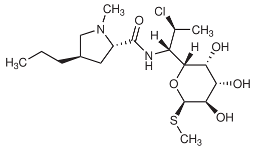
2	ambroxol PC3 DOWN	8.13255E-12	$C_{13}H_{18}Br_2N_2O$	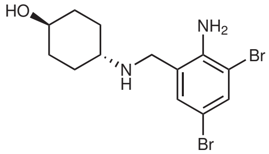
3	puromycin PC3 DOWN	1.00631E-7	$C_{22}H_{29}N_7O_5$	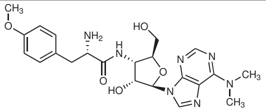
4	Metformin hydrochloride	1.28238E-7	$C_4H_{11}N_5$	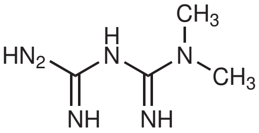
5	VITAMIN E CTD 00006994	7.38319E-7	$C_{29}H_{50}O_2$	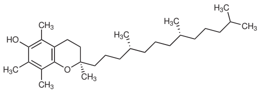
6	paclitaxel PC3 DOWN	2.43941E-6	$C_{47}H_{51}NO_{14}$	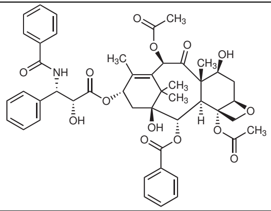
7	Carfilzomib	2.43941E-6	$C_{40}H_{57}N_5O_7$	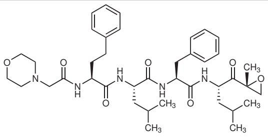
8	SC-560	2.43941E-6	$C_{17}H_{12}ClF_3N_2O$	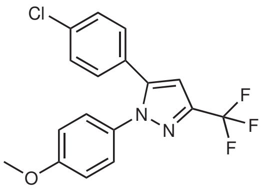
9	amikacin	2.43941E-6	$C_{22}H_{43}N_5O_{13}$	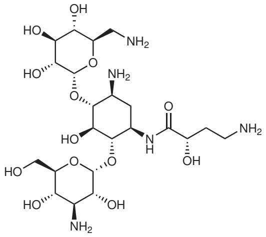
10	Bortezomib	1.173e-7	$C_{19}H25BN_4O_4$	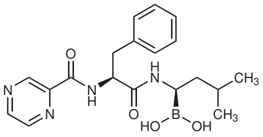

Supplementary Note 19: The utilization of self-attention, BERT, and residual networks in the task of RBP binding sites predictions.

Self-attention mechanisms have gained popularity across various fields for their ability to highlight important features in sequences while downplaying irrelevant ones. In the context of protein-RNA interactions, the self-attention model enable the focus on key nucleotides and their interactions. Several end-to-end models have already incorporated self-attention mechanisms; for instance, Wang et al. proposed SA-NET (56), which encodes nucleotide sequences into 4-mer tokens and identifies binding sites through a self-attention layer. Similarly, Pan et al. introduced CRMSNet (57), a deep learning model that leverages convolution and residual multi-head self-attention blocks to predict RBPs' binding sites on RNA sequence. These methods excessively rely on the self-attention mechanism, leading to an insufficient diversity of feature extraction and a high demand for data for training (58). To address this limitation, BERT, short for "Bidirectional Encoder Representations from Transformers", was proposed by designing a deep learning model based on self-attention mechanism. Its strength lies in understanding the contextual relationships of words within sentences by analyzing the text data bidirectionally. Given the similarity between nucleotide sequences and textual sequences, BERT has been adopted for the identification of RBP binding sites. For example, Yamada et al. proposed BERT-RBP as a model to predict RNA–RBP interactions by adapting the BERT architecture pre-trained on a human reference genome (59). The principal shortcoming of this kind of BERT-based method lies in the fact that its fine-tuning process cannot significantly optimize the parameters of the BERT model, leading to inaccuracies in capturing the binding characteristics of RBPs, particularly under dynamic cellular conditions. Nevertheless, the pre-training process of BERT, which involves learning from a vast amount of transcriptome data, empowers it to comprehensively grasp the contextual relationships within the genome. Therefore, such advantages make BERT an attractive option to be leveraged as a generative model for creating dynamic contextual representations of nucleotide sequences. Several end-to-end deep learning models based on this dynamic contextual representations have been developed, including HCRNet (60), CircSSNN (61), and JLCRB (62). However, most of these end-to-end methods were designed to identify RBP binding sites on circular RNAs under a singular cellular condition. To the best of our knowledge, the dynamic contextual representation of nucleotide sequences has not yet been characterized for dynamic prediction of RBP binding sites on linear RNA (across different cell lines or in vivo tissues), which motivates our further research in this field.

ResNets, initially introduced in the field of computer vision (63), have brought about a revolutionary impact. Now, these ResNets are applied to the field of RBP binding sites identification. ResNets can solve the vanishing gradient problem common in deep neural networks, allowing for the successful training of very deep networks. Numerous end-to-end models have adopted the residual structure to predict RBP binding sites, including CRBPDL (64), CRMSS (65), iCircRBP-DHN (66), and CircSSNN. Unfortunately, these methods are primarily focused on predicting RBP binding sites on circular RNAs in specific cell lines, limiting their broader applicability. Furthermore, one common aspect in these methods, including PrismNet, is the use of a basic ResNet structure, which integrates multiple features before inputting them into the residual network. This basic architecture possesses a risk as the amalgamation of features might lead to an inability to distinguish between sequence-specific and structural binding characteristics, thus restricting the performance of RBP binding site identification. To mitigate this risk, we propose a hierarchical multi-scale residual structure, comprising a parallel, dual-pathway framework that can extract the latent features of RNA sequences and secondary structures separately, ensuring the optimal performance in our unique dynamic prediction task. Moreover, the multi-scale structure enables HDRNet to learn binding patterns across varying sequence lengths.

Supplementary References

1. Tianqi Chen and Carlos Guestrin. Xgboost: A scalable tree boosting system. In *Proceedings of the 22nd acm sigkdd international conference on knowledge discovery and data mining*, pages 785–794, 2016.
2. Leo Breiman. Random forests. *Machine learning*, 45(1):5–32, 2001.
3. David W Hosmer Jr, Stanley Lemeshow, and Rodney X Sturdivant. *Applied logistic regression*, volume 398. John Wiley & Sons, 2013.
4. Anil K Jain, Jianchang Mao, and K Moidin Mohiuddin. Artificial neural networks: A tutorial. *Computer*, 29(3):31–44, 1996.
5. Pierre Geurts, Damien Ernst, and Louis Wehenkel. Extremely randomized trees. *Machine learning*, 63:3–42, 2006.
6. Bianca Zadrozny and Charles Elkan. Transforming classifier scores into accurate multiclass probability estimates. In *Proceedings of the eighth ACM SIGKDD international conference on Knowledge discovery and data mining*, pages 694–699, 2002.
7. Hajer Kamel, Dhahir Abdulah, and Jamal M Al-Tuwaijari. Cancer classification using gaussian naive bayes algorithm. In *2019 International Engineering Conference (IEC)*, pages 165–170. IEEE, 2019.
8. Fabian Pedregosa, Gaël Varoquaux, Alexandre Gramfort, Vincent Michel, Bertrand Thirion, Olivier Grisel, Mathieu Blondel, Peter Prettenhofer, Ron Weiss, Vincent Dubourg, et al. Scikit-learn: Machine learning in python. *the Journal of machine Learning research*, 12:2825–2830, 2011.
9. Andreas R Gruber, Ronny Lorenz, Stephan H Bernhart, Richard Neuböck, and Ivo L Hofacker. The vienna rna websuite. *Nucleic acids research*, 36(suppl_2):W70–W74, 2008.
10. Silvi Rouskin, Meghan Zubradt, Stefan Washietl, Manolis Kellis, and Jonathan S Weissman. Genome-wide probing of rna structure reveals active unfolding of mrna structures in vivo. *Nature*, 505 (7485):701–705, 2014.
11. Meghan Zubradt, Paromita Gupta, Sitara Persad, Alan M Lambowitz, Jonathan S Weissman, and Silvi Rouskin. Dms-mapseq for genome-wide or targeted rna structure probing in vivo. *Nature methods*, 14(1):75–82, 2017.
12. Damian Szklarczyk, Annika L Gable, David Lyon, Alexander Junge, Stefan Wyder, Jaime Huerta-Cepas, Milan Simonovic, Nadezhda T Doncheva, John H Morris, Peer Bork, et al. String v11: protein–protein association networks with increased coverage, supporting functional discovery in genome-wide experimental datasets. *Nucleic acids research*, 47(D1):D607–D613, 2019.
13. Eric L Van Nostrand, Gabriel A Pratt, Alexander A Shishkin, Chelsea Gelboin-Burkhart, Mark Y Fang, Balaji Sundararaman, Steven M Blue, Thai B Nguyen, Christine Surka, Keri Elkins, et al. Robust transcriptome-wide discovery of rna-binding protein binding sites with enhanced clip (eclip). *Nature methods*, 13(6):508–514, 2016.
14. Filippo Tamanini, Rob Willemsen, Leontine van Unen, Carola Bontekoe, Hans Galjaard, Ben A Oostra, and André T Hoogeveen. Differential expression of fmr1, fxr1 and fxr2 proteins in human brain and testis. *Human molecular genetics*, 6(8):1315–1322, 1997.

15. Yan Zhang, J Patrick O'Connor, Mikiko C Siomi, Sudha Srinivasan, Amalia Dutra, Robert L Nussbaum, and Gideon Dreyfuss. The fragile x mental retardation syndrome protein interacts with novel homologs fxr1 and fxr2. *The EMBO journal*, 14(21):5358–5366, 1995.
16. Corinne M Spencer, Ekaterina Serysheva, Lisa A Yuva-Paylor, Ben A Oostra, David L Nelson, and Richard Paylor. Exaggerated behavioral phenotypes in fmr1/fxr2 double knockout mice reveal a functional genetic interaction between fragile x-related proteins. *Human molecular genetics*, 15(12):1984–1994, 2006.
17. Carola JM Bontekoe, Kellie L McIlwain, Ingeborg M Nieuwenhuizen, Lisa A Yuva-Paylor, Anna Nellis, Rob Willemsen, Zhe Fang, Laura Kirkpatrick, Cathy E Bakker, Robin McAninch, et al. Knockout mouse model for fxr2: a model for mental retardation. *Human molecular genetics*, 11(5):487–498, 2002.
18. Lei Sun, Kui Xu, Wenze Huang, Yucheng T Yang, Pan Li, Lei Tang, Tuanlin Xiong, and Qiangfeng Cliff Zhang. Predicting dynamic cellular protein–rna interactions by deep learning using in vivo rna structures. *Cell research*, 31(5):495–516, 2021.
19. Eric L Van Nostrand, Peter Freese, Gabriel A Pratt, Xiaofeng Wang, Xintao Wei, Rui Xiao, Steven M Blue, Jia-Yu Chen, Neal AL Cody, Daniel Dominguez, et al. A large-scale binding and functional map of human rna-binding proteins. *Nature*, 583(7818):711–719, 2020.
20. Marianne Goodwin, Apoorva Mohan, Ranjan Batra, Kuang-Yung Lee, Konstantinos Charizanis, Francisco José Fernández Gómez, Sabiha Eddarkaoui, Nicolas Sergeant, Luc Buée, Takashi Kimura, et al. Mbnl sequestration by toxic rnas and rna misprocessing in the myotonic dystrophy brain. *Cell reports*, 12(7):1159–1168, 2015.
21. Yumin Zhu, Gang Xu, Yucheng T Yang, Zhiyu Xu, Xinduo Chen, Binbin Shi, Daoxin Xie, Zhi John Lu, and Pengyuan Wang. Postar2: deciphering the post-transcriptional regulatory logics. *Nucleic acids research*, 47(D1):D203–D211, 2019.
22. Eric T Wang, Neal AL Cody, Sonali Jog, Michela Biancolella, Thomas T Wang, Daniel J Treacy, Shujun Luo, Gary P Schroth, David E Housman, Sita Reddy, et al. Transcriptome-wide regulation of pre-mrna splicing and mrna localization by muscleblind proteins. *Cell*, 150(4):710–724, 2012.
23. Chantal Sellier, Fernande Freyermuth, Ricardos Tabet, Tuan Tran, Fang He, Frank Ruffenach, Violaine Alunni, Herve Moine, Christelle Thibault, Adeline Page, et al. Sequestration of drosha and dgcr8 by expanded cgg rna repeats alters microrna processing in fragile x-associated tremor/ataxia syndrome. *Cell reports*, 3(3):869–880, 2013.
24. Aaron R Haeusler, Christopher J Donnelly, Goran Periz, Eric AJ Simko, Patrick G Shaw, Min-Sik Kim, Nicholas J Maragakis, Juan C Troncoso, Akhilesh Pandey, Rita Sattler, et al. C9orf72 nucleotide repeat structures initiate molecular cascades of disease. *Nature*, 507(7491):195–200, 2014.
25. Scott M Lundberg and Su-In Lee. A unified approach to interpreting model predictions. *Advances in neural information processing systems*, 30, 2017.
26. Christian Von Mering, Lars J Jensen, Berend Snel, Sean D Hooper, Markus Krupp, Mathilde Foglierini, Nelly Jouffre, Martijn A Huynen, and Peer Bork. String: known and predicted protein–protein associations, integrated and transferred across organisms. *Nucleic acids research*, 33(suppl_1):D433–D437, 2005.
27. Taiwen Li, Jingxin Fu, Zexian Zeng, David Cohen, Jing Li, Qianming Chen, Bo Li, and X Shirley Liu. Timer2. 0 for analysis of tumor-infiltrating immune cells. *Nucleic acids research*, 48(W1): W509–W514, 2020.
28. Guangyan Zhou, Othman Soufan, Jessica Ewald, Robert EW Hancock, Niladri Basu, and Jianguo Xia. Networkanalyst 3.0: a visual analytics platform for comprehensive gene expression profiling and meta-analysis. *Nucleic acids research*, 47(W1):W234–W241, 2019.
29. Li-Xia Dong, Hai-Lan Bao, Yan-Yun Zhang, Yu Liu, Guo-Wei Zhang, and Feng-Mao An. Microrna-16-5p/btg2 axis affects neurological function, autophagy and apoptosis of hippocampal neurons in alzheimer's disease. *Brain Research Bulletin*, 175:254–262, 2021.
30. YH Taguchi and Hsiuying Wang. Exploring microrna biomarkers for parkinson's disease from mrna expression profiles. *Cells*, 7(12):245, 2018.
31. Nairita Ahsan Faruqui, Durdana Hossain Prium, Sadrina Afrin Mowna, Tanjim Ishraq Rahaman, Arundhati Roy Dutta, and Mst Farjana Akter. Identification of common molecular signatures shared between alzheimer's and parkinson's diseases and therapeutic agents exploration: An integrated genomics approach. *bioRxiv*, pages 2020–12, 2021.
32. Penny J Norsworthy, Andrew GB Thompson, Tze H Mok, Fernando Guntoro, Luke C Dabin, Akin Nihat, Ross W Paterson, Jonathan M Schott, John Collinge, Simon Mead, et al. A blood mirna signature associates with sporadic creutzfeldt-jakob disease diagnosis. *Nature communications*, 11(1):1–11, 2020.
33. Radhika Raheja, Keren Regev, Brian C Healy, Maria Antonietta Mazzola, Vanessa Beynon, Felipe Von Glehn, Anu Paul, Camilo Diaz-Cruz, Taha Gholipour, Bonnie I Glanz, et al. Correlating serum micrornas and clinical parameters in amyotrophic lateral sclerosis. *Muscle & nerve*, 58(2):261–269, 2018.
34. Sandra Van der Auwera, Sabine Ameling, Matthias Nauck, Henry Völzke, Uwe Völker, and Hans J Grabe. Plasma circulating micro-rnas associated with alexithymia reflect a high overlap on neuropsychiatric outcomes. *Journal of Affective Disorders*, 305:206–212, 2022.
35. Fatemeh Zadehbagheri, Ebrahim Hosseini, Zahra Bagheri-Hosseinabadi, Hossein Moradi Rekabdarkolaee, and Iman Sadeghi. Profiling of mirnas in serum of children with attention-deficit hyperactivity disorder shows significant alterations. *Journal of psychiatric research*, 109:185–192, 2019.
36. Pengxiang Li, Ying Xu, Baiping Wang, Jiali Huang, and Qiang Li. mir-34a-5p and mir-125b-5p attenuate aβ-induced neurotoxicity through targeting bace1. *Journal of the neurological sciences*, 413:116793, 2020.
37. Norikazu Hara, Masataka Kikuchi, Akinori Miyashita, Hiroyuki Hatsuta, Yuko Saito, Kensaku Kasuga, Shigeo Murayama, Takeshi Ikeuchi, and Ryozo Kuwano. Serum microrna mir-501-3p as a potential biomarker related to the progression of alzheimer's disease. *Acta neuropathologica communications*, 5(1):1–9, 2017.
38. Pavan Kumar, Zoltan Dezso, Crystal MacKenzie, Judy Oestreicher, Sergei Agoulnik, Michael Byrne, Francois Bernier, Mamoru Yanagimachi, Ken Aoshima, and Yoshiya Oda. Circulating mirna biomarkers for alzheimer's disease. *PloS one*, 8(7):e69807, 2013.
39. Mario Malerba and Beatrice Ragnoli. Ambroxol in the 21st century: pharmacological and clinical update. *Expert opinion on drug metabolism & toxicology*, 4(8):1119–1129, 2008.
40. Matthew E Gegg and Anthony HV Schapira. The role of glucocerebrosidase in parkinson disease pathogenesis. *The FEBS journal*, 285(19):3591–3603, 2018.
41. CRA Silveira, J MacKinley, K Coleman, Z Li, E Finger, R Bartha, SA Morrow, J Wells, M Borrie, RG Tirona, et al. Ambroxol as a novel disease-modifying treatment for parkinson's disease dementia: Protocol for a single-centre, randomized, double-blind, placebo-controlled trial. *BMC neurology*, 19:1–10, 2019.
42. Alisdair McNeill, Joana Magalhaes, Chengguo Shen, Kai-Yin Chau, Derralyn Hughes, Atul Mehta, Tom Foltynie, J Mark Cooper, Andrey Y Abramov, Matthew Gegg, et al. Ambroxol improves lysosomal biochemistry in glucocerebrosidase mutation-linked parkinson disease cells. *Brain*, 137(5):1481–1495, 2014.
43. Stephen Mullin, Laura Smith, Katherine Lee, Gayle D'Souza, Philip Woodgate, Josh Elflein, Jenny Hällqvist, Marco Toffoli, Adam Streeter, Joanne Hosking, et al. Ambroxol for the treatment of patients with parkinson disease with and without glucocerebrosidase gene mutations: a nonrandomized, noncontrolled trial. *JAMA neurology*, 77(4):427–434, 2020.
44. Alexandra Bouscary, Cyril Quessada, Althéa Mosbach, Noëlle Callizot, Michael Spedding, Jean-Philippe Loeffler, and Alexandre Henriques. Ambroxol hydrochloride improves motor functions and extends survival in a mouse model of familial amyotrophic lateral sclerosis. *Frontiers in pharmacology*, 10:883, 2019.
45. Jing Wang, Denis Gallagher, Loren M DeVito, Gonzalo I Cancino, David Tsui, Ling He, Gordon M Keller, Paul W Frankland, David R Kaplan, and Freda D Miller. Metformin activates an atypical pkc-cbp pathway to promote neurogenesis and enhance spatial memory formation. *Cell stem cell*, 11(1):23–35, 2012.
46. Jared M Campbell, Matthew D Stephenson, Barbora De Courten, Ian Chapman, Susan M Bellman, and Edoardo Aromataris. Metformin use associated with reduced risk of dementia in patients with diabetes: a systematic review and meta-analysis. *Journal of Alzheimer's Disease*, 65(4):1225–1236, 2018.
47. Amit Gupta, Bharti Bisht, and Chinmoy Sankar Dey. Peripheral insulin-sensitizer drug metformin ameliorates neuronal insulin resistance and alzheimer's-like changes. *Neuropharmacology*, 60(6): 910–920, 2011.
48. Nikita Katila, Sunil Bhurtel, Sina Shadfar, Sunil Srivastav, Sabita Neupane, Uttam Ojha, Gil-Saeng Jeong, and Dong-Young Choi. Metformin lowers α-synuclein phosphorylation and upregulates neurotrophic factor in the mptp mouse model of parkinson's disease. *Neuropharmacology*, 125:396–407, 2017.
49. David S Siegel, Thomas Martin, Michael Wang, Ravi Vij, Andrzej J Jakubowiak, Sagar Lonial, Suzanne Trudel, Vishal Kukreti, Nizar Bahlis, Melissa Alsina, et al. A phase 2 study of single-agent carfilzomib (px-171-003-a1) in patients with relapsed and refractory multiple myeloma. *Blood, The Journal of the American Society of Hematology*, 120(14):2817–2825, 2012.
50. Qiuyang Zheng, Timothy Huang, Lishan Zhang, Ying Zhou, Hong Luo, Huaxi Xu, and Xin Wang. Dysregulation of ubiquitin-proteasome system in neurodegenerative diseases. *Frontiers in aging neuroscience*, 8:303, 2016.
51. Zaira Ortega and Jose J Lucas. Ubiquitin–proteasome system involvement in huntington's disease. *Frontiers in molecular neuroscience*, 7:77, 2014.
52. Chris McKinnon and Sarah J Tabrizi. The ubiquitin-proteasome system in neurodegeneration. *Antioxidants & redox signaling*, 21(17):2302–2321, 2014.
53. Christopher A Ross and Michelle A Poirier. Protein aggregation and neurodegenerative disease. *Nature medicine*, 10(Suppl 7):S10–S17, 2004.
54. Jianing Jin, Li Xue, Xinling Bai, Xiaona Zhang, Qingwu Tian, and Anmu Xie. Association between epidermal growth factor receptor gene polymorphisms and susceptibility to parkinson's disease. *Neuroscience Letters*, 736:135273, 2020.
55. Pusheng Quan, Kai Wang, Shi Yan, Shirong Wen, Chengqun Wei, Xinyu Zhang, Jingwei Cao, and Lifen Yao. Integrated network analysis identifying potential novel drug candidates and targets for parkinson's disease. *Scientific Reports*, 11(1):1–9, 2021.
56. Xinyi Wang, Mingyang Zhang, Chunlin Long, Lin Yao, and Min Zhu. Self-attention based neural network for predicting rna-protein binding sites. *IEEE/ACM Transactions on Computational Biology and Bioinformatics*, 20(2):1469–1479, 2022.
57. Zhengsen Pan, Shusen Zhou, Hailin Zou, Chanjuan Liu, Mujun Zang, Tong Liu, and Qingjun Wang. Crmsnet: A deep learning model that uses convolution and residual multi-head self-attention block to predict rbps for rna sequence. *Proteins: Structure, Function, and Bioinformatics*, 2023.
58. Haixin Lv, Jinglong Chen, Tongyang Pan, Tianci Zhang, Yong Feng, and Shen Liu. Attention mechanism in intelligent fault diagnosis of machinery: A review of technique and application. *Measurement*, page 111594, 2022.
59. Keisuke Yamada and Michiaki Hamada. Prediction of rna–protein interactions using a nucleotide language model. *Bioinformatics Advances*, 2(1):vbac023, 2022.
60. Yuning Yang, Zilong Hou, Yansong Wang, Hongli Ma, Pingping Sun, Zhiqiang Ma, Ka-Chun Wong, and Xiangtao Li. Hcrnet: high-throughput circrna-binding event identification from clip-seq data using deep temporal convolutional network. *Briefings in Bioinformatics*, 23(2), 2022.
61. Chao Cao, Shuhong Yang, Mengli Li, and Chungui Li. Circssnn: circrna-binding site prediction via sequence self-attention neural networks with pre-normalization. *BMC bioinformatics*, 24(1):220, 2023.

62. Xiuquan Du and Zhigang Xue. Jlcrb: A unified multi-view-based joint representation learning for circrna binding sites prediction. *Journal of Biomedical Informatics*, 136:104231, 2022.
63. Kaiming He, Xiangyu Zhang, Shaoqing Ren, and Jian Sun. Deep residual learning for image recognition. In *Proceedings of the IEEE conference on computer vision and pattern recognition*, pages 770–778, 2016.
64. Mengting Niu, Quan Zou, and Chen Lin. Crbpdl: Identification of circrna-rbp interaction sites using an ensemble neural network approach. *PLoS computational biology*, 18(1):e1009798, 2022.
65. Lishen Zhang, Chengqian Lu, Min Zeng, Yaohang Li, and Jianxin Wang. Crmss: predicting circrna-rbp binding sites based on multi-scale characterizing sequence and structure features. *Briefings in Bioinformatics*, 24(1):bbac530, 2023.
66. Yuning Yang, Zilong Hou, Zhiqiang Ma, Xiangtao Li, and Ka-Chun Wong. icircrbp-dhn: identification of circrna-rbp interaction sites using deep hierarchical network. *Briefings in Bioinformatics*, 22 (4):bbaa274, 2021.
