# Supplementary material for: Dynamic characterization and interpretation for protein-RNA interactions across diverse cellular conditions using HDRNet
Source: Nat Commun. 2023 Oct 26;14:6824. doi: 10.1038/s41467-023-42547-1 (PMC10603054; doi:10.1038/s41467-023-42547-1)
Supplement: Supplementary file 3 — Reporting Summary [file 41467_2023_42547_MOESM3_ESM.pdf]

nature portfolio

Corresponding author(s): Xiangtao Li, Ka-Chun Wong

Last updated by author(s): 2023-Sep-14

Reporting Summary

Nature Portfolio wishes to improve the reproducibility of the work that we publish. This form provides structure for consistency and transparency in reporting. For further information on Nature Portfolio policies, see our Editorial Policies and the Editorial Policy Checklist.

Statistics

For all statistical analyses, confirm that the following items are present in the figure legend, table legend, main text, or Methods section.

n/a	Confirmed	
☐	☒	The exact sample size (*n*) for each experimental group/condition, given as a discrete number and unit of measurement
☒	☐	A statement on whether measurements were taken from distinct samples or whether the same sample was measured repeatedly
☐	☒	The statistical test(s) used AND whether they are one- or two-sided<br>*Only common tests should be described solely by name; describe more complex techniques in the Methods section.*
☒	☐	A description of all covariates tested
☒	☐	A description of any assumptions or corrections, such as tests of normality and adjustment for multiple comparisons
☐	☒	A full description of the statistical parameters including central tendency (e.g. means) or other basic estimates (e.g. regression coefficient) AND variation (e.g. standard deviation) or associated estimates of uncertainty (e.g. confidence intervals)
☐	☒	For null hypothesis testing, the test statistic (e.g. *F*, *t*, *r*) with confidence intervals, effect sizes, degrees of freedom and *P* value noted<br>*Give P values as exact values whenever suitable.*
☒	☐	For Bayesian analysis, information on the choice of priors and Markov chain Monte Carlo settings
☒	☐	For hierarchical and complex designs, identification of the appropriate level for tests and full reporting of outcomes
☒	☐	Estimates of effect sizes (e.g. Cohen's *d*, Pearson's *r*), indicating how they were calculated

Our web collection on statistics for biologists contains articles on many of the points above.

Software and code

Policy information about availability of computer code

Data collection	No software was used for data collection.
Data analysis	We used R package ClusterProfile (v3.18.1), DESeq2 (v1.40.2) and Python package STRING (v11.5), Cytoscape (v3.9.0), and Timer (v2.0) for data analysis. NetworkAnalyst web (v3.0) was employed for data analysis. We adopted RNAfold from ViennaRNA package (v2.6.3) for RNA structure prediction and visualization and bedtools (v2.30.0) to obtain RNA sequences for tissue data. Source code, documentation, and instruction for HDRNet package (v1.0.0.1) is available at GitHub repository: https://github.com/zhuhr213/HDRNet and HDRNet is also released at Zenodo database at https://doi.org/10.5281/zenodo.8341649.

For manuscripts utilizing custom algorithms or software that are central to the research but not yet described in published literature, software must be made available to editors and reviewers. We strongly encourage code deposition in a community repository (e.g. GitHub). See the Nature Portfolio guidelines for submitting code & software for further information.

Data

Policy information about availability of data

All manuscripts must include a data availability statement. This statement should provide the following information, where applicable:

- Accession codes, unique identifiers, or web links for publicly available datasets
- A description of any restrictions on data availability
- For clinical datasets or third party data, please ensure that the statement adheres to our policy

We collected 261 RBP binding sites datasets for cell lines across multiple databases, including 172 RBPs constructed using the same flag-marked technique in K562, HepG2, HEK293, HEK293T, HeLa and H9 cell lines. These datasets include 65 CLLP-seq datasets for 61 RBPs from POSTAR database \cite{zhu2019postar2} and 196 eCLIP datasets for 111 RBPs from the ENCODE project \cite{van2020large}. These data have been deposited in \cite{sun2021predicting}. In terms of the RBP binding

data in tissues, the processed MBNL2 (Muscleblind Like Splicing Regulator 2) binding peak data in human brain tissues \cite{goodwin2015mbnl} were available in POSTAR database \cite{zhu2019postar2} under accession code \href{https://www.ncbi.nlm.nih.gov/geo/query/acc.cgi?acc=GSE68890}{GSE68890}; the DGCR8 and HNRNPU binding data in human adrenal gland were collected from ENCODE project \cite{van2020large}; and the MBNL1 (Muscleblind Like Splicing Regulator 1) binding data in brain, heart, muscle, and myoblasts from mice were obtained from \cite{wang2012transcriptome} under accession code \href{https://www.ncbi.nlm.nih.gov/geo/query/acc.cgi?acc=GSE39911}{GSE39911}. We have deposited these datasets in FigShare database (\href{https://doi.org/10.6084/m9.figshare.24132423}{https://doi.org/10.6084/m9.figshare.24132423}) \cite{Zhu2023}. These datasets can also be downloaded from our HDRNet web-server at \href{http://www.aibio-lab.com:5050/}{http://www.aibio-lab.com:5050/}. {\color{blue}Source data are provided with this paper.

Field-specific reporting

Please select the one below that is the best fit for your research. If you are not sure, read the appropriate sections before making your selection.

[x] Life sciences   [ ] Behavioural & social sciences   [ ] Ecological, evolutionary & environmental sciences

For a reference copy of the document with all sections, see nature.com/documents/nr-reporting-summary-flat.pdf

Life sciences study design

All studies must disclose on these points even when the disclosure is negative.

Sample size	Sample data were obtained from public repositories. Sample size was not predetermined and was determined by previous published datasets which were used for our study. Chosen datasets were used to evaluate HDRNet.
Data exclusions	No data were excluded from the analysis.
Replication	The experiments were performed three times. All attempts at replication were successful.
Randomization	Not applicable - no experimental groups were assigned.
Blinding	Blinding is not relevant to our study since we report computational analysis as main finding.

Reporting for specific materials, systems and methods

We require information from authors about some types of materials, experimental systems and methods used in many studies. Here, indicate whether each material, system or method listed is relevant to your study. If you are not sure if a list item applies to your research, read the appropriate section before selecting a response.

Materials & experimental systems

n/a	Involved in the study	
[x]	[ ]	Antibodies
[x]	[ ]	Eukaryotic cell lines
[x]	[ ]	Palaeontology and archaeology
[x]	[ ]	Animals and other organisms
[x]	[ ]	Human research participants
[x]	[ ]	Clinical data
[x]	[ ]	Dual use research of concern

Methods

n/a	Involved in the study	
[x]	[ ]	ChIP-seq
[x]	[ ]	Flow cytometry
[x]	[ ]	MRI-based neuroimaging
